# Closing Yield Gaps: How Sustainable Can We Be?

**DOI:** 10.1371/journal.pone.0129487

**Published:** 2015-06-17

**Authors:** Prajal Pradhan, Günther Fischer, Harrij van Velthuizen, Dominik E. Reusser, Juergen P. Kropp

**Affiliations:** 1 Potsdam Institute for Climate Impact Research, Potsdam, Germany; 2 International Institute for Applied Systems Analysis, Laxenburg, Austria; 3 Dept. of Geo- and Environmental Sciences, University of Potsdam, Potsdam, Germany; Instituto de Agricultura Sostenible (CSIC), SPAIN

## Abstract

Global food production needs to be increased by 60–110% between 2005 and 2050 to meet growing food and feed demand. Intensification and/or expansion of agriculture are the two main options available to meet the growing crop demands. Land conversion to expand cultivated land increases GHG emissions and impacts biodiversity and ecosystem services. Closing yield gaps to attain potential yields may be a viable option to increase the global crop production. Traditional methods of agricultural intensification often have negative externalities. Therefore, there is a need to explore location-specific methods of sustainable agricultural intensification. We identified regions where the achievement of potential crop calorie production on currently cultivated land will meet the present and future food demand based on scenario analyses considering population growth and changes in dietary habits. By closing yield gaps in the current irrigated and rain-fed cultivated land, about 24% and 80% more crop calories can respectively be produced compared to 2000. Most countries will reach food self-sufficiency or improve their current food self-sufficiency levels if potential crop production levels are achieved. As a novel approach, we defined specific input and agricultural management strategies required to achieve the potential production by overcoming biophysical and socioeconomic constraints causing yield gaps. The management strategies include: fertilizers, pesticides, advanced soil management, land improvement, management strategies coping with weather induced yield variability, and improving market accessibility. Finally, we estimated the required fertilizers (N, P_2_O_5_, and K_2_O) to attain the potential yields. Globally, N-fertilizer application needs to increase by 45–73%, P_2_O_5_-fertilizer by 22–46%, and K_2_O-fertilizer by 2–3 times compared to the year 2010 to attain potential crop production. The sustainability of such agricultural intensification largely depends on the way management strategies for closing yield gaps are chosen and implemented.

## Introduction

The global crop demand for human consumption and livestock feed is expected to increase by 60–110% between 2005 and 2050 [1, 2]. Changing dietary habits towards more affluent diets consisting of a larger share of animal products, vegetable oils, and sugar-sweeteners [3] as well as the growing world population are the main drivers of the global crop demand [4]. Increasing crop demand can be fulfilled either by expanding cropland and harvested area, and/or by increasing crop yields. Between 1985 and 2005, the global crop production has increased by 28% of which only 8% came from the expansion of cropland and harvested area, and 20% from increased crop yields [5]. However, yields of the four major crops (maize, rice, wheat, and soybean) have either stagnated or collapsed over the period of 1961–2008 across 24% to 39% of their cultivated areas globally [6]. Yield growths per year of these crops over the same period is substantially less than the annual growth rate of 2.4% that will be required for doubling crop production by 2050 [7]. Presently, crop yields vary across regions even within the same climatic zones [8]. These variations in crop yields are related to market accessibility, purchasing power/income, agricultural work force, and terrain factors [9], besides water and fertilizer management [10]. However, closing yield gaps will enhance food self-sufficiency (FSS) and enable food security at local, regional, and global scales [11].

In the future, the global mean biophysical potential crop yield is likely to be reduced due to climate change compared to an unchanging climate scenario [12]. Agriculture is one of the sectors, which is highly vulnerable to climate change and climate extremes which will increase the prevalence of agricultural production constraints, e.g., heat and water stress, and changes in pest and disease ranges. The effects of climate change will be significant on low-input agricultural systems characterized by large yield gaps due to traditional management approaches [13, 14]. Furthermore, productivity of high-input agricultural systems (e.g., irrigated farming) may also be affected because of decreased water availability and increased crop water demand due to rise in temperature. Nevertheless, in some regions, crop production may benefit from moderate global warming [15], for example, North European crop production could benefit from climate change and increased atmospheric CO_2_ [16–18].

Crop production requires various inputs, of which nutrients (e.g., nitrogen, phosphorus, and potassium) and water are crucial. At the same time, associated water management and application of fertilizers and other agrochemicals may cause environmental stress and loss of biodiversity [19]. The agricultural sector consumes approximately 70% of the global water withdrawal [20]. Globally, about 24% of the total cultivated land is irrigated (around 310 million hectares), which in return produces about 33% of the global crop production [21]. The agricultural sector directly contributes about 10% to 12% of the total anthropogenic greenhouse gas (GHG) emissions [22]. Indirectly, agriculture additionally shares emissions related to land conversion that is responsible for 12% of the total GHG emissions [22]. Cropland expansion has negative impacts on biodiversity and ecosystem services [23] and hence, may not be a sustainable option to increase crop production. Agricultural intensification increases crop yields, but may reduce GHG emissions by unit of production due to avoided land conversion [24]. Nevertheless, the traditional way of intensifying agriculture also has negative effects, e.g., nutrient loss and ecosystem deterioration [25]. Therefore, we need to explore ways to sustainably intensify current agriculture systems considering a broad range of potential management interventions that have been accessed in a more focused way [9, 10]. These management interventions should address the future food demand, closing crop yield gaps, and the minimization of environmental stress [1, 4, 11].

Consequently, the general objective of this study is to conceive location specific agricultural management and input options required for closing yield gaps to increase the crop production, meeting the present and future food demand. Here, crop yield gaps are defined as differences between the modeled potential attainable yields under high-input and advanced management assumptions and the downscaled achieved crop yields in 2000. More precisely, this study has the three specific objectives. Since every location may not need to attain potential yields to meet its food demand if export is excluded, our first objective is to identify regions where the closure of yield gaps matters in terms of reducing food deficits and improving food self-sufficiency (FSS). The second objective is to figure out agricultural inputs and management interventions that are needed to close yield gaps across various regions through exploring spatially explicit factors causing yield gaps. The third objective is to quantify required nutrients to attain potential yields in different locations.

## Materials and Methods

Data used for this study is obtained from the Global Agro-ecological Zones (GAEZv3.0), the detailed methodology of which is presented in the GAEZv3.0 model documentation [26]. In short, based on principles of land evaluation, GAEZv3.0 estimates crop production potential described as the agronomically possible upper limit of crop yields for individual crops under given agro-climatic, soil, and terrain conditions for a specific level of agricultural inputs and management conditions. For crop production potential, GAEZv3.0 defines three generic input levels (low, intermediate, and high input levels). Under a low-input level, the farming system is considered largely subsistence and labor intensive based on traditional management, using local crop varieties. Under an intermediate-input level, the farming system is considered partly market oriented with a mixture of subsistence based and commercial scale production. Under a high-input level, the farming system is assumed to be mainly commercial agriculture with mechanized management, using adequate nutrients, agro-chemicals, and high yielding crop varieties. Additionally, to supplement potential yield information, GAEZv3.0 also provides downscaled crop yields and area harvested for the year 2000.

### Production Gaps and Calorie Deficits

We defined crop production gaps as a ratio between the potential and the current crop calorie production. To estimate the calorie productions, we used data on current and potential crop yields, and area harvested in 2000 for 19 crop types from GAEZv3.0 [26] and nutritive factors for converting crop mass into calories from FAO [27] (S1 Table). GAEZv3.0 provides in a global raster grid of 5 arc minutes resolution information on both current and potential crop yields for two types of water supply (irrigated and rain-fed), and potential crop yields for the three input levels. We estimated the potential crop calorie production using crop yield data under high-input levels (S1 Text).

We analyzed crop calorie deficits based on the demand and supply of crop calories. The demand side consists of human vegetal product consumption and crop-based feed provided to livestock, which were calculated from gridded feed data [28], countrywide per capita vegetal product intake [29], and gridded population data [30] for the year 2000. The supply side includes crop calorie production that was derived from GAEZv3.0 [26] (S1 Text). Since agricultural production constraints and management vary with agro-climatic conditions, crop calorie deficits and production gap analysis was conducted in sub-national moisture regime units [26]. The seven moisture regime categories used here are: hyper-arid, arid, dry semi-arid, moist semi-arid, sub-humid, humid, and per-humid. We identified regions with crop calorie deficits considering the current and the potential crop calorie production (S1 Text). Afterwards, we classified these regions into six groups based on prevalence and depth of crop production gaps and crop calorie deficits (S1 Text). By doing so, we located regions where closing production gaps results in FSS or significantly reduces calorie deficit status in a single global map.

Scenario analysis for two scenarios (scenario A and B) were used to identify regions where closing the production gaps matters and ensures future FSS by 2050 applying the above described method. Scenario A in which population changes but dietary patterns remain constant at in the year 2000 level, is a baseline scenario. Scenario B accounts for country specific changes in dietary patterns in addition to the population growth; maintaining a minimum calorie intake of 2,535 kcal/cap/day, representing the average for high calorie diets [3]. In this way, we accounted for changes in population [31] and dietary patterns [3] that drive future food and feed demand [1, 2, 28], and progress on closing crop yield gaps that influence future food and feed supply [10, 12] (S1 Text).

### Yield Gap Factors

We observed substantially larger yield gaps for rain-fed farming than for irrigated farming (S1 Fig). Globally, rain-fed farming covers 74% of cultivated land. So far it produces only 44% of the potential calorie production while irrigated farming has attained 60% of the potential calorie production. Hence, we focus this analysis on rain-fed cultivated land as it has a larger potential of additional crop production by closing yield gaps than irrigated land.

**Fig 1:**
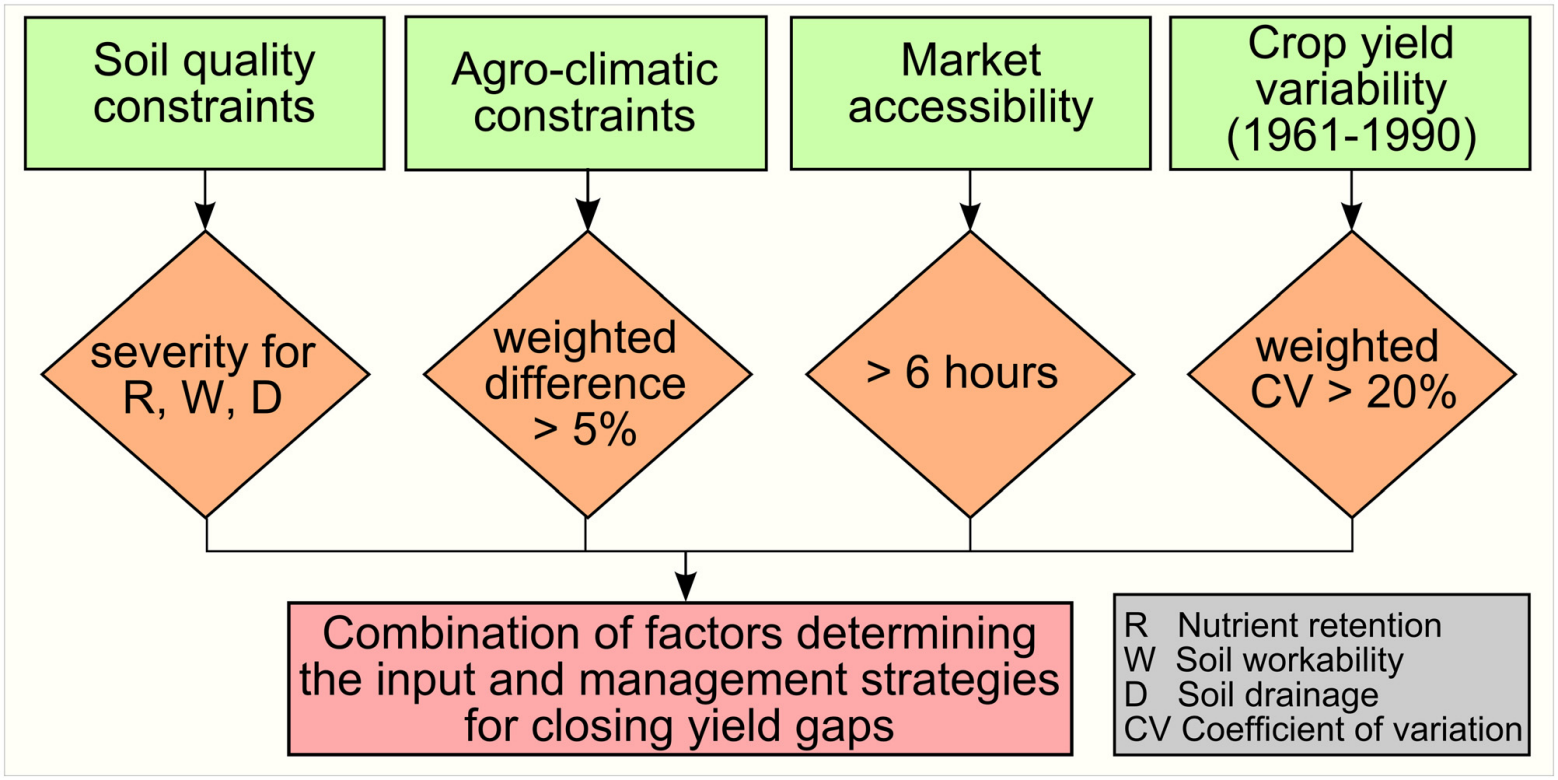
Flow chart showing the procedure used to design agricultural input and management strategies required to close yield gaps. The light green boxes represent the input data obtained from GAEZv3.0 [26]. The applied procedures are symbolized by the light orange diamonds, which are explained in S2 Text. The light red box shows the obtained result.

A number of biophysical and socioeconomic factors puts constraints on crop yields [32, 33], resulting in yield gaps that can be tackled with adequate agricultural input and management (Fig 1). Initially, we analyzed the biophysical factors that can be overcome by shifting farming practices from traditional low-input to high-input advanced management. We started the analysis looking at agro-climatic constraints related to yield losses due to pests, diseases, weeds, and workability. The first three of the constraints can be reduced by improved pest management. However, the workability constraint related to weather conditions affecting the efficiency of farming operation (e.g., excessive wetness causing problems in harvesting and handling of crop products) is hard to tackle.

We obtained data on crop specific agro-climatic constraints for low and high input levels at a 5′ resolution from GAEZv3.0. The constraints are characterized through the attainable percentage of the crop yields. Crop yields are determined by radiation and temperature regimes and water availability for a specific input level. The attainable percentage of crop yields is higher for high-input level, closing crop yield gaps, compare to that for low-input level. This is because improved pest management can reduce agro-climatic constraints related to yield losses due to pests, diseases, and weeds. We estimated the difference between the agro-climatic constraints for low and high input levels. The differences were calculated for crops in the two major crop groups (cereals and roots-tubers) and averaged with weights based on harvested area. In the year 2000, these two crop groups combined contributed around 80% of the total crop calorie production. By this, we identified regions where agro-climatic constraints could be attenuated by shifting from low to high input farming based on the weighted difference between agro-climatic constraints larger than 5% (Fig 1 and S2 Text).

As the second factor, we identified regions where crop production is hampered by soil quality constraints. GAEZv3.0 differentiates seven soil qualities and classifies them into four spatially explicit categories: no or slight, moderate, severe, and very severe constraints. Among them, constraints related to three soil qualities (rooting conditions, excess salt, and toxicity) are difficult to overcome using high inputs. Moreover, nutrient availability is an essential soil quality to attain high yields and is assessed separately as described in the next section. Hence, we identified regions where constraints related to one or more of the remaining three soil qualities (nutrient retention capacity, soil drainage, and soil workability) are moderate to very severe. These are the regions where crop yields can be increased by soil and land management that improves the soil qualities.

Next, we attempted to capture socioeconomic factors playing important roles in closing yield gaps based on two indicators: yield variability and travel time to the nearest market. The yield variability due to weather conditions may make farmers reluctant to take risks in terms of input applications without which crop yield increments are difficult [34]. GAEZv3.0 provides data on the coefficient of variation of agro-climatically attainable yields for the baseline period of 1961–1990 [26]. We used this data for crops in two crop groups (cereals and roots-tubers) to estimate the weighted yield variations based on irrigated and rain-fed harvested area, and identified regions with overall year-to-year yield variations larger than 20% (S2 Text).

Travel time to the nearest market is an important factor in enhancing agricultural productivity as it determines farmers’ accessibility to inputs and influences market approachability for selling agricultural products. Consequently, we used spatially explicit accessibility data presenting travel time to the nearest market with a population of around 50,000 [26] to identify regions with a connecting time longer than 6 hours to markets. We used the traveling time of 6 hours as threshold because the numbers of smaller cities and towns decreases subsequently with increase in the travel time beyond 6 hours [35].

We integrated the information from the four constraints (agro-climate constraints, soil quantity constraints, weather induced yield variability, and market accessibility) by identifying regions with similar dominant constraints. For each combination of dominant constraints, we identified management strategies needed to tackle the prevailing single or multiple constraints (Fig 1 and S2 Text). These management strategies are a novel approach to overcome and reduce yield gaps considering the biophysical and socioeconomic factors that have an impact on crop yields. For attaining high-input yields, implementation of these strategies is needed in addition to application of adequate nutrients and use of high yielding crop varieties. Moreover, we estimated additional crop calories that can be produced by implementing these strategies based on the differences between the current and the high-input potential crop calorie production.

### Required Nutrients

Nutrient management plays a crucial role in closing yield gaps [10]. To obtain crop yields constantly above the low-input levels, fertilizer application is needed in addition to natural nutrient regeneration. Hence, we quantified the amount of fertilizers required to attain high-input potential crop yields considering differences in crop production under low and high input levels. As nutrients absorbed by crops are stored in crop products (e.g., grain) and residues (e.g., straw), we considered differences between yields and residues of the 16 crop types from GAEZv3.0 for high and low input levels, also accounting for fallow period requirements. GAEZv3.0 provides crop specific fallow period requirements for high and low input levels by crop group, by soil type and by climatic condition. A low-input farming system requires a longer fallow period for natural nutrient regeneration that is substituted by fertilizer application in high-input agriculture, shortening the fallow period requirement. We calculated residue for a crop type based on its yield and harvest index [26]. Afterwards, we estimated the amount of additional crop nutrient uptake in crop yields and residues while attaining high-input potential yields. For this, we multiplied the crop harvested area by the differences in crop yields and residues between low and high input levels, and by the crop specific nutrients uptake in yield and in residue, respectively (S1 Table). We assumed that both crop products and residues are removed from the fields. Hence, nutrient removal that has to be replenished by fertilizers (organic or chemical), is equal to the total nutrient uptake in yields and residues. Since fertilizers applied to crops may get lost due to leaching and volatilization, the total fertilizers required also varies depending on fertilizer application efficiencies. By this, we estimated the quantities of three macro-nutrients required (N, P_2_O_5_, and K_2_O) to achieve the potential high-input yields (see S3 Text) assuming that micro nutrient constraints are tackled in fertilizer specific nutrient compositions.

## Results

### Focus Regions for Closing Yield Gaps

**Fig 2:**
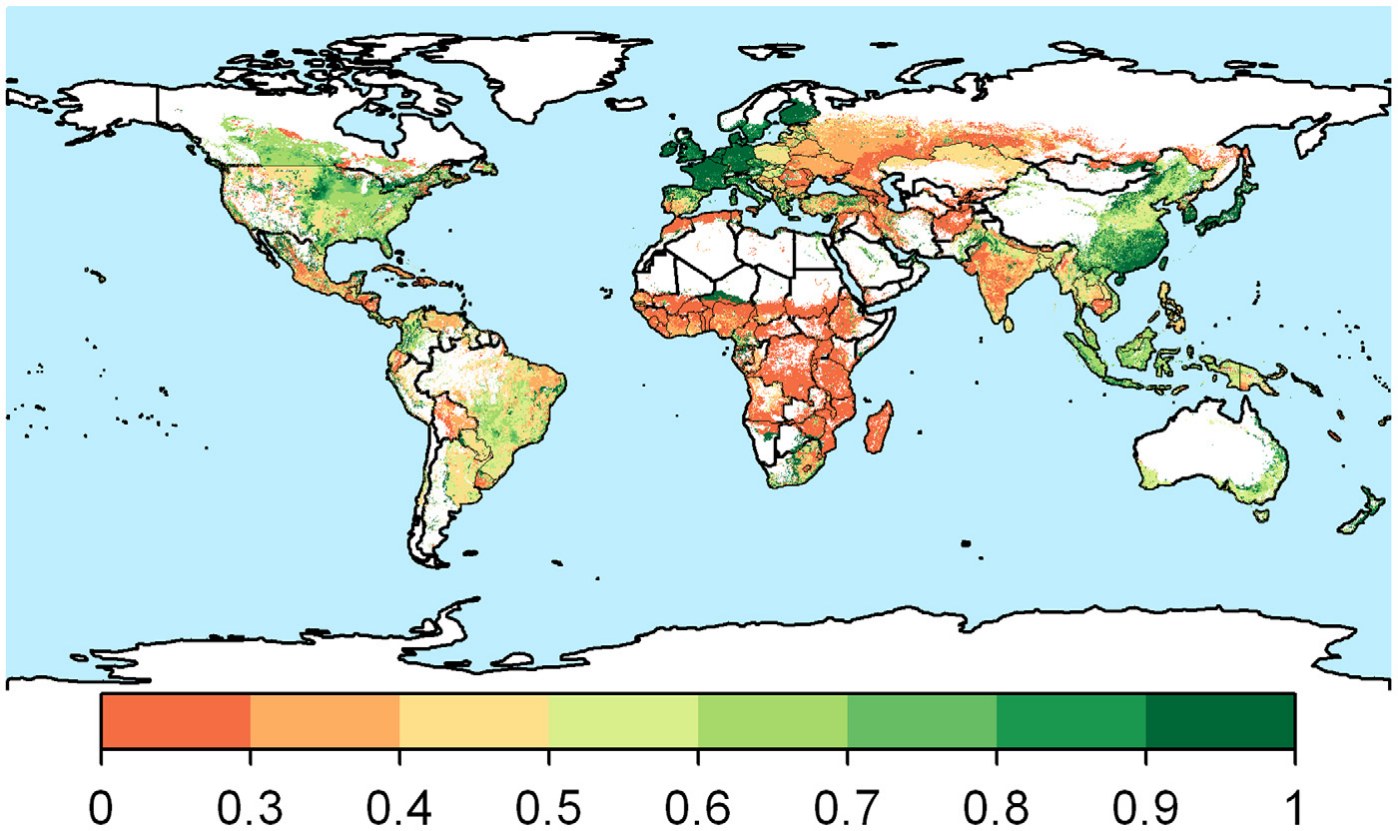
Location specific ratio of high-input crop calorie production attained in 2000. A ratio of 1 represents regions that have achieved their high-input crop calorie production.

We found that modern agriculture practices have enabled us to produce globally about 50% more crop calories than can potentially be produced by farming under low-input levels. However, this achievement varies spatially and is mainly concentrated in the parts of Oceania, West Europe, North Europe, North America, South America, and South-East Asia (S2 Fig). The current global crop calorie production can be doubled by adapting available high-input agriculture practices in the present cultivated land with the present cropping patterns. So far only North and West European countries have almost met their high-input potential crop calorie production (Fig 2). Countries in North America, South-East Asia, and Oceania have mostly achieved more than 60% of their potential production. Countries in Africa and East Europe are at the lower end with the achievement of less than 40%.

**Fig 3:**
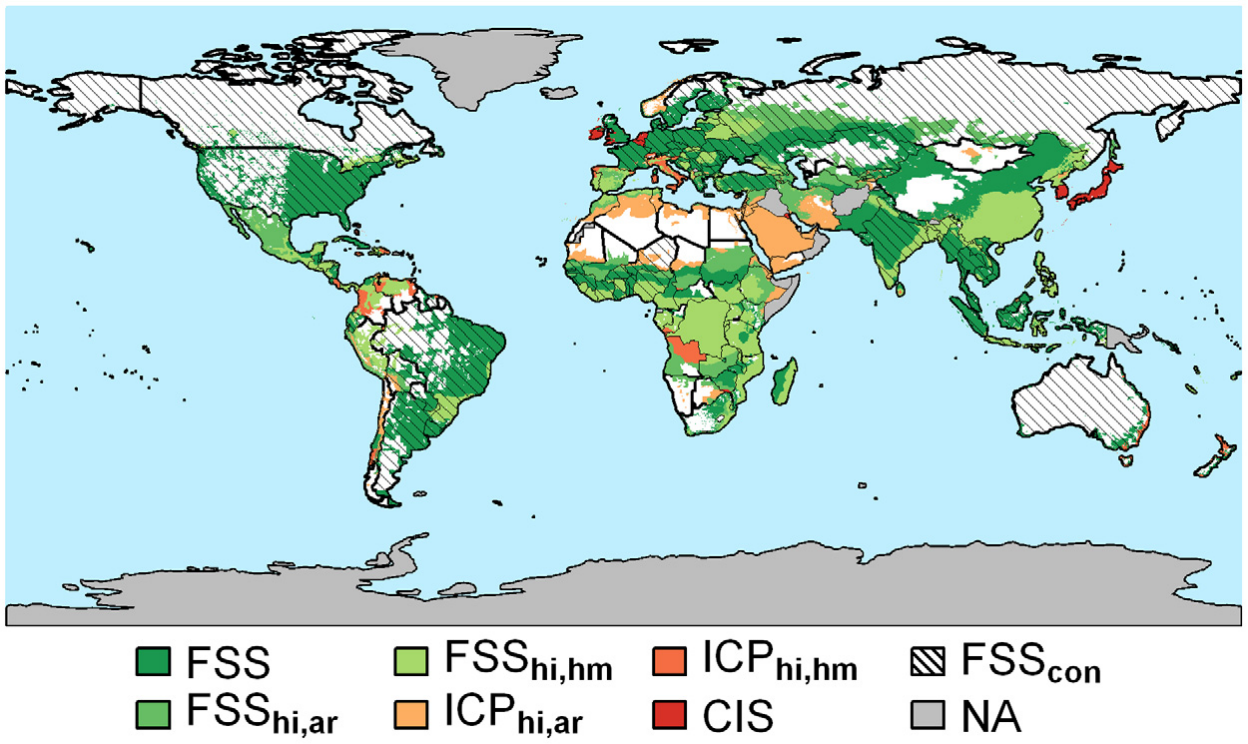
Regions that can achieve food self-sufficiency (FSS) based on their current crop production, have the potential to be food self-sufficient by attaining high-input (hi) yields, can only increase crop production (ICP) or almost attained high-input yields but are crop insufficient (CIS) by country (con) and by moisture regime (arid (ar) and humid (hm)) for 2000. Since agricultural production constraints and agricultural management vary with agro-climatic conditions, the results are presented by country moisture regime going beyond national scales. NA represents regions with missing data.

Most African countries could produce enough food to meet their consumption requirements by achieving their potential yields (Fig 3). In many North and South American, European, and Asia-Pacific countries, the current food production meets their calorie requirements (S3 Fig). However, for some countries even achieving potentials would be insufficient to meet their food demand due to poor agricultural land resource conditions. For example, although countries in arid regions, such as the Middle East, may increase crop production and close yield gaps, these countries cannot become food self-sufficient. Some countries (e.g., Japan) have approximately achieved high-input potential yields, but are not food self-sufficient. This is also related to limited cultivable land availability and population. Other countries (such as the United States, India, and Brazil) are food self-sufficient at the national level but not in all climate zones.

**Fig 4:**
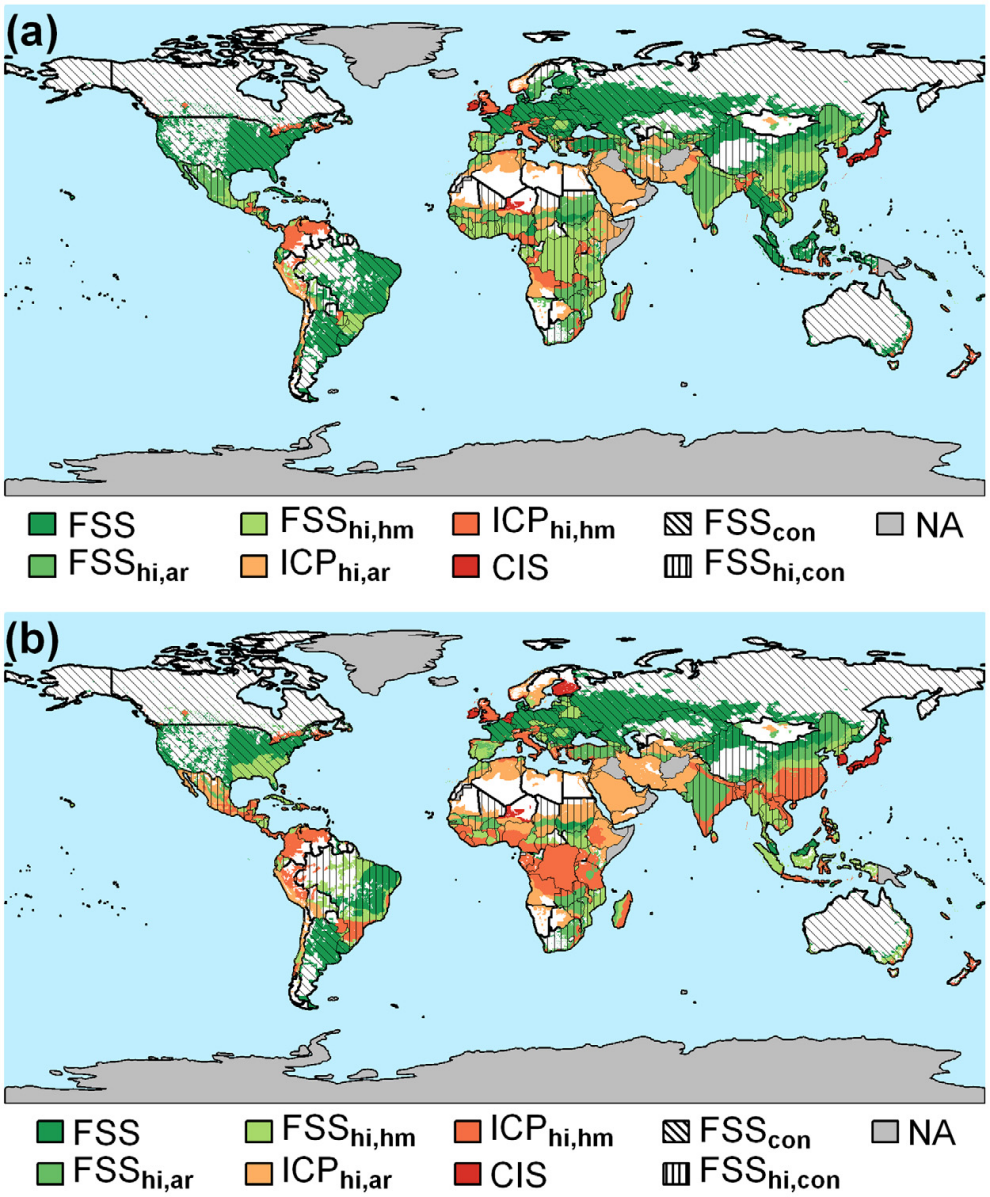
Regions that can achieve food self-sufficiency (FSS) based on their current crop production have the potential to be food self-sufficient by attaining high-input (hi) yields, can only increase crop production (ICP) or almost attained high-input yields but are crop insufficient (CIS) by country (con) and by moisture regime (arid (ar) and humid (hm)) considering two scenarios for 2050. (a) changes in population only, and (b) changes in population and dietary patterns. Since agricultural production constraints and agricultural management vary with agro-climatic conditions, the results are presented by country moisture regime going beyond national scales. NA represents regions with missing data.

By 2050, some countries (such as the United States, Canada, Brazil, France, and Germany) can be food self-sufficient even based on their current crop production, provided their dietary patterns remain unchanged (Scenario A, Fig 4a), whereas many African and Asian countries can be food self-sufficient when realizing high-input potentials. In contrast, some countries like Pakistan and Bangladesh cannot achieve FSS by closing yield gaps due to high population growth projected by 2050. In Africa, we find only a few countries that can produce sufficient food to feed a growing population by closing yield gaps under the assumptions of shifting dietary habits by 2050 (Scenario B, Fig 4b). This implies yield gap closure can only decrease food insecurity and cannot reach FSS in the majority of African countries. Changes in dietary habits may not cause large differences in most developed countries which already have high consumption levels which are not likely to increase significantly by 2050. Countries in transition like India and China may see regional variations in FSS as a result of changing dietary habits on top of population growth. Nevertheless, closing yield gaps can enable food security in most countries globally. This requires the application of inputs as well as sound management for tackling the constraints causing such yield gaps, which may vary spatially. We will elaborate on this in the next section.

### Input and Management Strategies

#### Biophysical and Socioeconomic Constraints

Constraints that must be dealt to design the input and management strategies for closing yield gaps are: agro-climate related pest, disease, and weed constraints, soil related constraints, weather induced yield variability as well as market accessibility. Climate related pest, disease, and weed constraints are prevalent in both humid regions, e.g., parts of Indonesia, China, and South America, and arid regions, e.g., parts of India, Sahel, and South Africa (S4a Fig). In these regions, agro-climatic pest, disease, and weed constraints are responsible for up to 30% of the weighted differences between high and low input crop yields (S2 Text). Apart from poor nutrition status, soil constraints that must be overcome to achieve potential yields are nutrient retention capacity, soil drainage, and soil workability. Nutrient retention problems are widespread in Sub-Saharan Africa, East Europe, and the eastern part of the United States (S4b Fig). Poor soil drainage generally affects dryland crop (e.g., wheat) production and is particularly prevalent in the Baltic States (e.g., Estonia, Latvia, and Lithuania), in some regions in North and South America, and some coastal countries such as Bangladesh. Soil workability will be a prevalent constraint in, for example, parts of India, Ethiopia, and Mexico. In some regions multiple soil constraints need to be dealt with, e.g., parts of Nepal, Myanmar, Thailand, Cambodia, Vietnam, and Laos (S4b Fig).

Apart from above biophysical factors, socioeconomic constraints also need to be tackled to close yield gaps. The first indicator we analyzed, yield variability shows a higher yield variation in semi-arid zones worldwide, e.g., within the United States, Argentina, Angola, Namibia, South-Africa, Russia, Kazakhstan, India, China, and Australia (S4c Fig). The observed yield variability is mainly due to year by year fluctuations in weather conditions (e.g., precipitation). A second socioeconomic indicator analyzed, travel time to the nearest market, shows that it takes more than six hours to reach the nearest market with a population of around 50,000 from a large portion of presently cultivated land in Africa, South-East Asia, and Central Asia (S2 Table). This means that farmers of these lands have limited access to the market for buying agricultural inputs (e.g., fertilizers, good quality seeds, etc.) and to sell their production surplus. This is frequently coupled with poor access to agricultural extension services providing up-to-date knowledge on agricultural practices to farmers.

#### Need for Intervention

To achieve potential crop yields, specific input and management interventions are needed that tackle the constraints described above. Regions with prevailing agro-climatic pest, disease, and weed constraints require integrated pest management using agro-chemicals and/or biological controls. So far integrated pest management has been poorly adopted in many developing countries [36] resulting in a tremendous yield losses [37].

Soil constraints that can be partly overcome include poor nutrient status. The poor nutrient status of soils can be cured by applying an adequate type and amount of organic and/or chemical fertilizers, but a prerequisite is that soil nutrient retention capacity is sufficient. On large scales, it is hard to improve nutrition retention capacity. On local levels, appropriate soil management that helps to increase soil carbon content may improve the retention capacity. Poor soil drainage can be tackled by artificial drainage, however, this method is costly. Nevertheless, in countries producing mainly wetland crops like rice (e.g., Bangladesh) poor soil drainage is not necessarily a constraint. Another soil constraint, soil workability, when associated with soil type and soil texture (i.e., cracking clay soil) which cannot be handled with the traditional farming based on manual labor and animal power. Only machine utilization and careful timing of field operations can overcome this constraint. This is observable, e.g., in parts of South Asia, West Asia, and Europe. Moreover, North and West Europe have almost met their potential crop calorie production adopting high-input mechanized farming systems. Multiple soil constraints existing in various regions will best be addressed via high-input precision farming that can tackle more than one constraint at the same time [38].

Due to weather induced yield variability, farmers may be reluctant to take risks in terms of optimal application of inputs (e.g., fertilizers) resulting in lower yields. Measures to reduce risk of crop failure due to low precipitation (e.g., supplementary irrigation) and/or measures that encourage farmers to take risks (e.g., insurance schemes) may help to handle such sub-optimal farming to close yield gaps. In many regions the option of supplementary irrigation is not relevant due to lack of existing irrigation infrastructure or lack of accessible water resources. With climate change, measures to address such weather induced yield variability will increasingly be of relevance [39]. In regions with low market access, constructing and maintaining transport infrastructure may enable farmers to start closing parts of the yield gaps by using available inputs from the nearest market and benefit additional income from increased crop production and sales. For subsistence farming, access to market may be less relevant; for commercial farming it is a prerequisite.

#### Description of Derived Strategies and Their impacts

**Fig 5:**
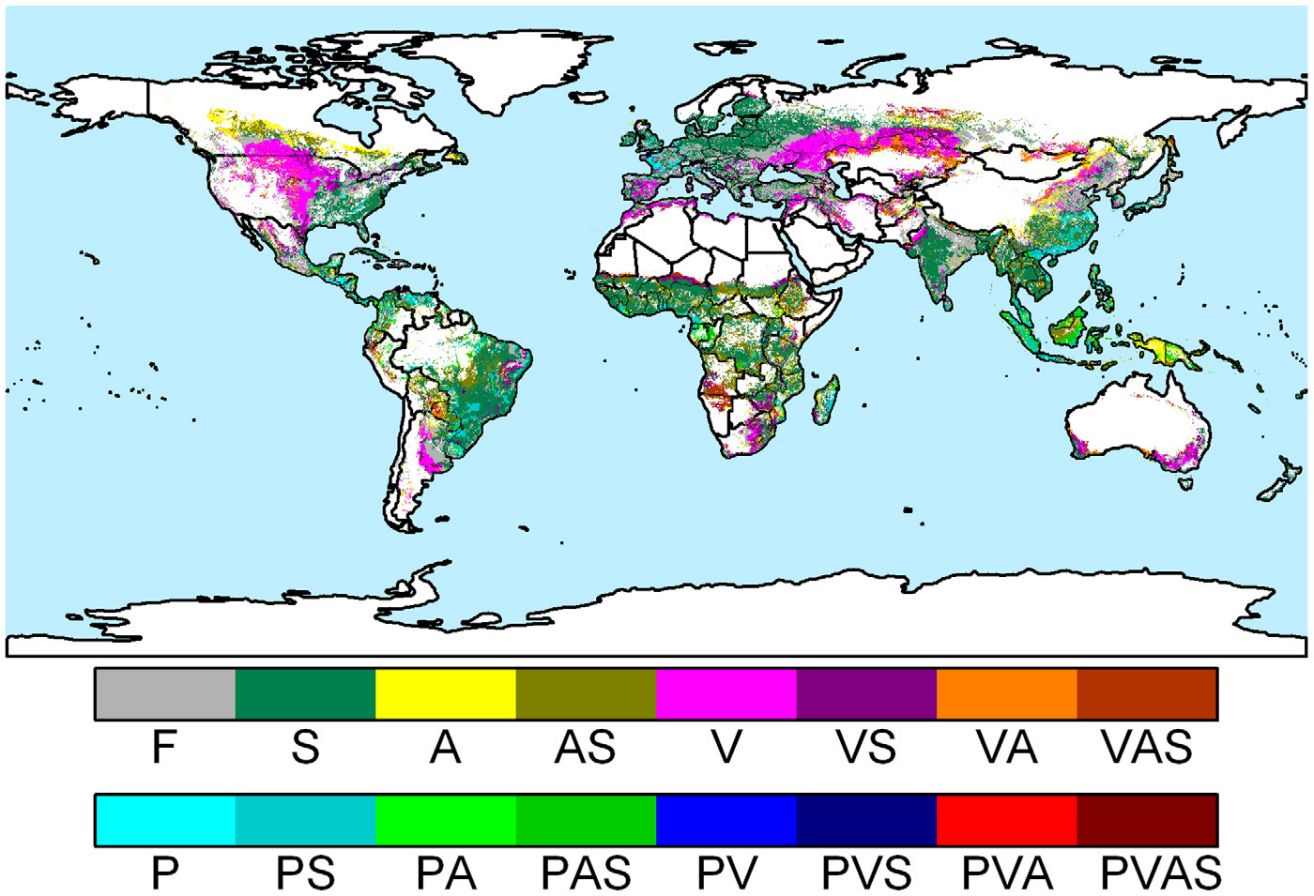
Location specific agricultural input and management strategies as required in different parts of the world to achieve high-input potential yields in addition to adequate fertilizer application (F). The strategies consist of soil quality management (S), managing accessibility to markets (A), weather induced yield variability management (V), and management of pests, diseases, and weeds (P). The different management strategies can have combinations of the individual elements (F, S, A, V, and P).

Biophysical and socioeconomic factors causing yield gaps vary spatially and multiple constraints may persist locally. This indicates the need for multiple input and management interventions to help overcome yield reducing constraints. We defined location specific input and management strategies as multiple required interventions in a location to tackle prevailing constraints for achieving potential yields. Fig 5 presents 16 different strategies required worldwide, which includes one or more of the following: pest, disease, and weed management (P), advanced soil management and land improvement (S), improving market accessibility (A), and management targeted on mitigating weather induced yield variability (V). Moreover, nutrient (fertilizer) management (F) and use of improved cultivars will go far in the regions without above-mentioned described constraints. Applying adequate nutrient management in currently available rain-fed cropland alone will produce an additional 1,700 trillion (1,700 × 10^12^) kcal/yr or an increase of around 20% globally compared to the year 2000 (Table 1). This amount will be sufficient to feed about 1.7 billion people with a daily diet of 2,800 kcal/cap/day. Parts of East Africa, West Africa, East Asia, West Asia, South Asia, North America, South America, and East Europe are among the regions having such potential (Fig 5). In these regions, a large amount of good and prime soil exist with none or little biophysical constraints (S2 Table). This lands can be rapidly used to increase crop yields with the sole investment of nutrient application. Another, additional 3,000 trillion kcal/yr of crops can be produced globally by overcoming prominent soil constraints (e.g., soil nutrition retention capacity, soil workability, and soil drainage) which prevail in South America, East Europe, South Asia, and West Africa. However, tackling these soil constraints requires large investments, e.g., building drainage infrastructure, general land improvement, and introducing farm mechanization. In Sub-Saharan Africa and South-East Asia, management strategies targeting multiple factors, e.g., a combination of advanced soil management with improved accessibility to the market, would potentially put an additional 500 trillion kcal/yr of crops in the world food market.

**Table 1:** Regional overview of additional crop calories that can be produced on rain-fed cultivated land by closing yield gaps with various management strategies in addition to adequate fertilizer application (F).

**Regions**	**F**	**S**	**A**	**AS**	**V**	**VS**	**VA**	**VAS**	**P**	**PS**	**PA**	**PAS**	**PV**	**PVS**	**PVA**	**PVAS**
	10^12^ kcal/yr															
**Africa**																
East Africa	71.6	280.9	37.0	114.3	9.9	43.0	6.6	18.4	8.5	27.7	2.0	5.0	1.4	1.4	0.2	0.3
Middle Africa	4.6	81.1	2.5	65.6	0.2	9.0	0.1	14.0	0.4	7.7	0.1	6.3	0.0	0.1	0.0	0.0
North Africa	40.5	79.7	7.7	37.2	32.8	59.2	0.8	4.5	0.2	0.3	0.6	0.8	0.0	0.0	0.0	0.0
South Africa	4.2	15.1	0.9	2.9	11.5	21.2	1.3	2.8	0.0	0.0	0.0	0.0	0.0	0.0	0.0	0.0
West Africa	94.7	560.0	18.4	91.5	0.1	10.9	0.1	5.4	1.9	25.9	0.2	3.9	0.0	0.0	0.0	0.0
**America**																
Caribbean	20.8	20.2	0.6	0.4	1.0	0.7	0.0	0.1	0.7	1.1	0.0	0.0	0.0	0.0	0.0	0.0
Central America	39.7	43.7	5.6	6.6	3.8	4.8	0.3	0.7	28.3	24.0	2.5	4.3	0.4	0.2	0.0	0.0
North America	164.8	190.9	7.0	6.2	247.6	78.4	9.4	1.5	0.7	7.3	0.0	0.2	0.0	0.1	0.0	0.0
South America	138.4	277.4	10.4	44.9	43.3	30.8	4.0	4.5	5.8	71.6	1.4	10.4	0.1	2.1	0.0	0.3
**Asia**																
Central Asia	3.5	1.1	0.5	0.2	29.9	5.5	18.4	5.5	0.0	0.0	0.0	0.0	0.0	0.0	0.0	0.0
East Asia	140.8	130.7	18.9	19.2	23.2	9.5	2.9	0.9	5.5	31.2	0.9	1.5	0.1	0.1	0.0	0.0
South Asia	322.1	527.8	13.4	32.6	30.1	42.8	4.2	6.3	3.3	8.3	0.1	0.7	0.0	0.1	0.0	0.1
S.-East Asia	52.3	213.4	24.6	58.3	0.5	0.8	0.2	1.1	34.1	125.0	11.5	76.3	0.1	0.3	0.1	0.7
West Asia	75.3	52.8	2.0	2.4	29.1	16.2	0.8	0.6	0.2	0.5	0.0	0.0	0.0	0.0	0.0	0.0
**Europe**																
East Europe	472.4	415.9	5.2	5.1	434.6	42.4	9.5	3.5	0.4	0.6	0.0	0.0	0.0	0.0	0.0	0.0
North Europe	10.7	45.2	0.0	0.3	0.0	0.0	0.0	0.0	0.7	1.9	0.0	0.0	0.0	0.0	0.0	0.0
South Europe	48.2	55.4	0.7	0.6	26.0	23.4	0.4	0.2	3.6	5.6	0.1	0.1	0.0	0.0	0.0	0.0
West Europe	13.5	13.3	0.0	0.1	0.1	0.2	0.0	0.0	2.8	2.0	0.0	0.0	0.1	0.0	0.0	0.0
**Oceania**																
Australia & New Zealand	5.6	8.3	0.4	1.7	21.7	20.8	3.6	5.0	0.1	0.1	0.0	0.0	0.0	0.0	0.0	0.0
**World**	**1,724**	**3,013**	**156**	**490**	**945**	**419**	**62**	**75**	**97**	**341**	**19**	**110**	**2.3**	**4.3**	**0.4**	**1.4**

The strategies consist of soil quality management (S), managing accessibility to markets (A), weather induced yield variability management (V), and management of pests, diseases, and weeds (P). The different management strategies can have combinations of the individual elements (F, S, A, V, and P). These values were estimated by summing up the differences between the current and the high-input potential crop calorie production by region and by the required management strategy.

### Required Fertilizers

**Fig 6:**
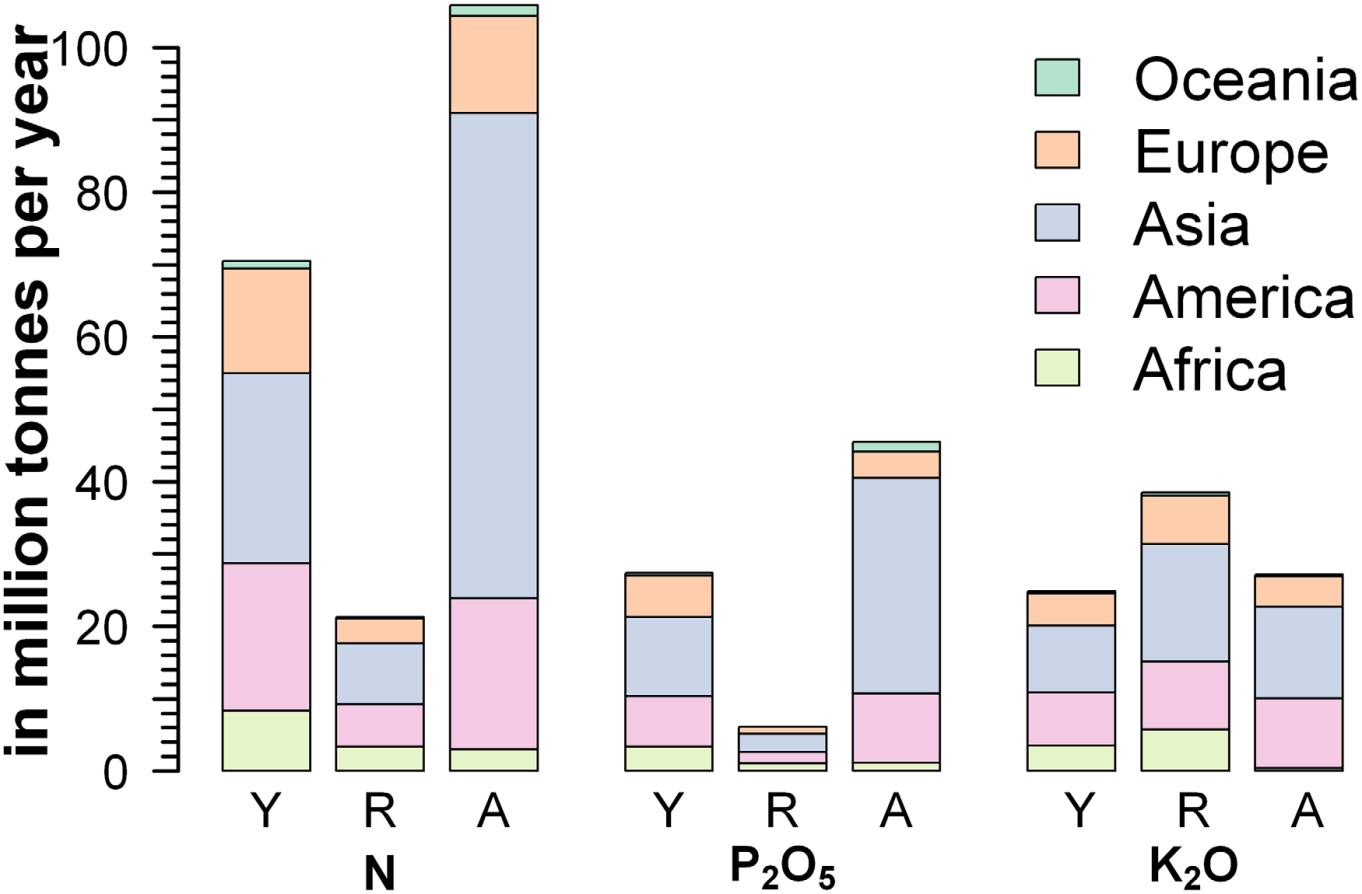
Additional amounts of macro-nutrients (N, P_2_O_5_, and K_2_O) uptake by crop yields (Y) and crop residues (R) by attaining high-input potential yields compared to that with low-input yields, and the amount of fertilizers applied (A) in the year 2010 on a continental scale.

Assuming that all other management options will result in achieving potential yields, sufficient nutrients need to be available. Globally, we estimated a net need of about 91.8 million tonnes of nitrogen fertilizers (N total nutrients) per year to replenish additional crop nutrient uptake while attaining the potential yields in the present cultivated land with the present cropping patterns (Fig 6). This nutrient need consists of uptake in crop yields and crop residues with respective values of 70.5 and 21.3 million tonnes/year. A substantial amount of fertilizer is consumed in the form of nutrient uptake in crop residues. Furthermore, a net amount of 33 million tonnes of phosphate fertilizers (P_2_O_5_ total nutrients) per year and 63 million tonnes of potash fertilizers (K_2_O total nutrients) per year is needed in addition. In comparison, 106, 45, and 27 million tonnes/year of N, P_2_O_5_, and K_2_O were applied in the year 2010 globally, respectively [29]. When considering a global fertilizer application efficiency of 50% to 60%, application of N fertilizers needs to be increased by 45% to 73%, P_2_O_5_ by 22% to 46%, and K_2_O by more than 2 to 3 times for attaining the potential yields compared to that of the year 2010. These required nutrients can be of organic and/or chemical origin, of which phosphorus and potassium are finite resources. Hence, closing the nutrient loop related to human sanitation is an option for providing parts of the required nutrients [40], which also reduces nutrient mining. Additionally, a high dependency on inorganic fertilizer may be problematic for achieving the potential yields as industrial fertilizer production is energy and GHG emission intensive.

Apart from increasing the amount of fertilizer application, enhancing its application efficiency, a ratio between required and applied fertilizer amount, is needed not only to attain the potential yields but also to reduce total fertilizer demand. This is important because access and inefficient use of chemical fertilizers increases major fluxes of the nutrient cycles for which we have already transgressed the planetary boundary [41]. Moreover, we find variation in fertilizer requirements and fertilizer application efficiencies globally (Fig 6 and S5 Fig). East Asia, South Asia, South East Asia, North Europe, South Europe, West Europe, and Oceania are the regions with a higher amount of fertilizer application, mainly N and P_2_O_5_, than the amount of crop nutrient uptake by attaining the high-input potential yields (S3 Table). Among these regions, North and West Europe have almost attained their potential yields with fertilizer application efficiency of about 60% and can be considered a frontier in this regard. In East Asia, the application efficiency is limited to 40%.

## Discussion

Globally, about 24% (2,200 × 10^12^ kcal/yr) and 80% (7,350 × 10^12^ kcal/yr) more crop calories can be produced compared to the total crop calorie produced in 2000 by closing yield gaps in current irrigated and rain-fed cultivated land, respectively. For some countries worldwide, closing their yield gaps is sufficient to meet their crop demand by 2050 considering population growth and dietary pattern changes. However, closing yield gaps is insufficient to meet the future crop demand for many African countries mainly when dietary changes are accounted for. For closing yield gaps, we need to implement various location specific agricultural input and management strategies. Adequate application of nutrients alone can increase crop calorie production by almost 20% whereas improvement of soil quality alone with adequate fertilizer application can generate an additional 30%. Moreover, application of N, P_2_O_5_, and K_2_O fertilizers needs to be increased by up to 70%, 50%, and 300% respectively, for attaining the potential yields compared to that of the year 2010.

With our approach we introduce several innovations in identifying required input and management options to close yield gaps. The first innovation is the use of calorie unit aggregating crop productions based on GAEZv3.0 model outputs to determine the potential crop calorie production and the yield gaps. Although a limited number of studies have presented crop production in units other than mass units [5, 42], estimation of yield gaps in calories is still missing. This is important as human dietary requirements are generally measured in calorific values. In addition, our estimation of the possibility to double the present global crop calories by closing yield gaps is higher (approx. 40%) than the estimates by other studies [5, 10]. This is because our study presents the upper bounds of crop production under optimal high-input agriculture. So far few regions have achieved these high-input crop yields [26]. However, other studies use observed yield variations across similar agro-climatic zones to determine the yield gaps [5, 8, 10].

The second innovation of this study concerns the combining of crop calorie production gaps with crop calorie deficits to identify the regions where closing yield gaps matter in terms of reduction or elimination of crop calorie deficits. For several countries e.g., the United States, Russia, Brazil, Australia, and countries in West Europe closing the present yield gaps is not required to ensure their present and future food self-sufficiency. This may in return suggest rolling back some cultivated land in these regions for extensive low-input agriculture to lower impacts associated with intensified agricultural practices. However, these countries are presently net food exporters [29]. If economically profitable and environmentally efficient, these countries may increase crop production for export and thereby further close their yield gaps. In most developing countries in Asia and Africa, achieving high-input potential yields will substantially reduce the present and future food deficits, improving national food self-sufficiency levels. Nevertheless, closing yield gaps is insufficient for many African countries, implying the need for cropland expansion and/or international agricultural trade to provide their future crop demand.

Third, we present a global picture as to what agricultural management strategies are required in different parts of the world to achieve the potential yields. A wide range of bio-physical and socioeconomic factors are to be tackled through the implementation of these management strategies that include soil quality management, management of pests, diseases, and weeds, yield variability management, and management of accessibility to markets. So far studies have either explained yield gaps using global biophysical and socio-economic data but not identified required strategies to close yield gaps [9] or focused only on nutrient and water management to close yield gaps not looking at other required strategies [10]. Application of adequate nutrients is a basis to increase crop productivity. However, this alone may not be enough to achieve potential crop production on a global scale. For example, we found that 980 million hectares of current rain-fed cultivated land, contributing a crop calorie growth of around 60%, requires the above mentioned additional inputs and management beyond extra nutrients. Hence, we present packages of the required management strategies instead of focusing on one or two measures for closing yield gaps. Furthermore, we quantified additional nutrient uptake by crops while attaining the high-input potential yields compared to the low-input yields, enabling us to estimate the amount of required fertilizers. This adds further uniqueness to our study. The amount of required fertilizers also depends on their application efficiencies and the ways in which crop residues are handled.

Although our study provides clear findings, as mentioned above, interpretation of the results requires an understanding of its limitations. First, our crop calorie deficit analysis is based on total crop production and consumption, which does not account for dietary compositions. Nevertheless, our study identifies regions where closing the yield gaps matters for self-sufficiency. This can be achieved by shifting dietary choices towards regional products and/or by focusing agricultural practices to meet regional demands.

Second, our yield gap analysis for the year 2000 may not reflect the current yield gaps, but most data on downscaled crop yields is available only for circa 2000. Additionally, we did not account for possible technological progress, shifting the potential yields ceiling in the future. Nevertheless, yield gaps exist even while considering potential yields under currently available technology. Furthermore, climate heterogeneity within length of growing period (LGP) used in GAEZv3.0 is relatively large, rising concern regarding use of GAEZv3.0 to estimate yield gaps [43]. However, GAEZv3.0 takes into account this heterogeneity by applying thermal suitability screening procedure while simulating crop yields. The screening procedure makes use of other agro-ecological zone schemes (e.g., thermal climatic conditions, permafrost conditions, temperature profiles, vernalization conditions, etc.) including LGP for testing the match of prevailing conditions with crops’ temperature requirements [26]. Therefore, LGP is only one out of a number of agro-ecological zone schemes used to simulate crop yields.

Third, we used a simplistic approach to design the agricultural management strategies looking at constraints that can be overcome by high-input farming as compared to traditional low-input agriculture. Since the present crop calorie production in most regions is between low and high input levels, it would be valuable to know the current management conditions for specifying management strategies more precisely. However, availability of such information in sub-national levels is limited mainly to fertilizer application [44].

Fourth, our study did not include one of the most relevant forces that will influence future agricultural production namely, climate change. In the future, climate change can increase crop yield variability and shift pest and disease ranges. This is a matter of ongoing studies. However, the approach used in this study can also be applied to investigate required input and management strategies for closing yield gaps under changing climatic conditions.

Summing up, our study provides an important contribution to the debate on agricultural intensification, presenting a global map of required management and input interventions to achieve the potential crop yields. Agricultural intensification, resulting in higher yields and more frequent harvests, not only demands massive inputs but also causes environmental stresses [19]. However, intensification and/or expansion of cultivated land are two main options available for increasing food production to meet growing demands. Cropland expansion is not feasible in all parts of the world because of uneven distribution of the limited suitable land for agriculture [45]. Moreover, agricultural expansion leading to deforestation and land-use change is a major source of GHG emissions [22]. Therefore, agricultural intensification paired with the reduction of environmental consequences may be a preferred option to increase world food production. The sustainability of intensified agriculture highly depends on measures and methods with which required agricultural management strategies are chosen and implemented. In the future, these management strategies will also need to focus on adapting and building resilience to climate change, advancing towards climate smart agriculture [46]. Hence, there is an urgent need to explore synergies between closing yield gaps and minimizing the agricultural production induced environmental stresses.

## Supporting Information

S1 Text Identification of Regions to Focus. (PDF)

S2 Text Identification of Management Strategies. (PDF)

S3 Text Nutrients Required. (PDF)

S1 Fig Location specific ratio of high-input crop calorie production attained in 2000 based on types of water supply. (a) rain-fed cultivated land, and (b) irrigated cultivated land. A ratio of 1 represents regions that have achieved their high-input crop calorie production.
(TIF)

S2 Fig Map depicting ratio between the current crop calorie production for 2000 and the low-input crop calorie production. The values greater than 1 represent regions with the current crop calorie production larger than the low-input calorie production.
(TIF)

S3 Fig Ratio between consumption and production of crop calories by country and by moisture regimes. The values greater than 1 represent regions with crop calorie consumption greater than crop calorie production. Since agricultural production constraints and agricultural management vary with agro-climatic conditions, the results are presented by country moisture regime going beyond national scales. NA represents regions with missing data.
(TIF)

S4 Fig Indicators used to distinguish yield reducing factors that could be overcome by shifting from low-input to high-input farming. (a) weighted difference between agro-climatic constraints factor in percentage for low-input and high-input farming, (b) location specific most severe soil quality constraints based on three soil qualities (nutrient retention capacity (R), soil drainage (D), and soil workability (W)), and (c) weighted coefficient of variation of agro-climatic yields in percentage.
(TIF)

S5 Fig Additional amount of macro-nutrients (in tonnes per pixel per year) uptake by crops in their yields and residues by attaining high input potential yields compared to that with low input yields. (a) nitrogen—N total nutrients, (b) phosphate—P_2_O_5_ total nutrients, and (c) potash—K_2_O total nutrients.
(TIF)

S1 Table List of crop types for which data on harvest area (*H* ^*j*^) (second column) and crops for which data on potential yield (*Y* ^*k*^) (thrid column) is provided by GAEZv3.0 along with their nutritive factors (*f* ^*j*^), conversion factor (*c* ^*k*^) for harvested weight to dry weight and nutrient uptakes from various sources compiled by authors. (PDF)

S2 Table Regional overview on area of rain-fed cultivated land where different inputs and management strategies in addition to adequate fertilizer application (F) are required to close the yield gaps. The strategies consist of soil quality management (S), managing accessibility to markets (A), weather induced yield variability management (V), and management of pests, diseases, and weeds (P). The different management strategies can have combinations of the individual elements (F, S, A, V, and P).
(PDF)

S3 Table Regional overview on additional amount of macro-nutrients (N, P_2_O_5_, and K_2_O) uptake by crop yields (Y) and crop residues (R) by attaining high input potential yields compared to that with low input yields, and regional overview on the amount of fertilizers applied in the year 2010. (PDF)
