# Supplementary material for: Closing Yield Gaps: How Sustainable Can We Be?
Source: PLoS One. 2015 Jun 17;10(6):e0129487. doi: 10.1371/journal.pone.0129487 (PMC4470636; doi:10.1371/journal.pone.0129487)
Supplement: S1 Text — (PDF) [file pone.0129487.s001.pdf]

S1 Text Identification of Regions to Focus

S1.1 Text Production Gaps

We used data on crop yields and crop harvest area from GAEZv3.0 [1] for calculating total crop calorie production. GAEZv3.0 provides information on the current and potential crop yields for two types of water supply (irrigated and rain-fed) and the potential yields for three input levels (low, intermediate, and high) in a global raster grid of 5 arc minutes resolution. GAEZv3.0 downscaled the current crop yields and area harvested on the grid for the year 2000 based on agricultural production statistics from the FAO and modeled the potential yields considering agricultural land resource conditions. Detailed methodology used to estimate the potential crop yields and to downscale the current crop yields is provided in the GAEZv3.0 model documentation [1]. Firstly, we calculated the current gridded crop calorie production (Ca) according to equation 1 below using the data on the current crop yield (Ya_r^j and Ya_i^j , j: a crop type, r: rain-fed, i: irrigated) and area harvested (H_r^j and H_i^j), and respective nutritive factors for converting crop mass into calories (f^j) from FAO [2] (S1 Table).

$$Ca = \sum_{j=1}^{n} \left(\left(Ya_r^j \times H_r^j + Ya_i^j \times H_i^j \right) \times f^i \right) \tag{1}$$

Secondly, we used data on potential yields [1] for estimating the potential crop calorie production. The potential crop yields are provided in t/ha in dry weight equivalents whereas the nutritive factors (f^j) used to convert crop mass into crop calories are in terms of harvested weight. Hence, we used conversion factors (c^k) to convert yields in dry weight into harvested weight [1]. The combination of the crop production based on rain-fed and irrigated agriculture obtained by multiplying the high-input potential yields (Yh_r^k and Yh_i^k) by their respective harvested area (H_r^j and H_i^j) provides the potential crop calorie production for high-input levels (Ch) (equation 2). As GAEZv3.0 provides data on irrigated crop yields only for intermediate and high input levels [1], we assumed that the combination of crop yields based on low-input rain-fed (Yl_r^k) and intermediate-input irrigated agriculture (Ym_i^k) provides the low-input crop calorie production (Cl) (equation 3). Data on potential yields are available for all individual crops but data on harvested area are provided only for the crop type instead of the individual crops. Hence, for each cell we used data for the individual crop with the highest yield among this crop type (nc: number of crops).

$$Ch = \sum_{j=1}^{n} \left(\left(max \left(\left\{ \frac{Yh_r^k}{c^k} \right\}_{k=1}^{nc} \right) \times H_r^j + max \left(\left\{ \frac{Yh_i^k}{c^k} \right\}_{k=1}^{nc} \right) \times H_i^j \right) \times f^j \right) \tag{2}$$

$$Cl = \sum_{j=1}^{n} \left(\left(max \left(\left\{ \frac{Yl_r^k}{c^k} \right\}_{k=1}^{nc} \right) \times H_r^j + max \left(\left\{ \frac{Ym_i^k}{c^k} \right\}_{k=1}^{nc} \right) \times H_i^j \right) \times f^j \right) \tag{3}$$

Both equations above sum up the 19 crop types $j = 1 \ldots n$, accounting for more than 90% of the global crop calories produced in the year 2000. Among 23 crop types considered by GAEZv3.0, we excluded non-food crop (e.g., cotton and fodder), stimulant cash crops (e.g., tea, coffee, and cacao) and crops under residual section for this analysis.

We defined crop calorie production gap of a region as a ratio between potential and current crop calorie production in the region (Gh). The ratio less than 1 shows the potential of the region to produce more crops. Since agricultural management varies across agro-climatic zones, regions for the production gap analyses were defined by country and by moisture regime (CMR symbolized by z, and x symbolized the raster cell) as represented by equation 4. Information on seven moisture regimes was derived from GAEZv3.0 data on the length of growing period. The seven moisture regimes are: hyper-arid (0 days growing period), arid (1-59 days growing period), dry semi-arid (60-119 days growing period), moist

semi-arid (120-179 days growing period), sub-humid (180-269 days growing period), humid (270-365 days growing period) and per-humid (365 days continuous growing period). The length of growing period represents the number of favorable days during the year for crop growth based on moisture availability and temperature [1].

$$Gh_z = \left(\sum_{x \in z} Ca_x\right) / \left(\sum_{x \in z} Ch_x\right) \tag{4}$$

S1.2 Text Calorie Deficits

We identified regions with crop calorie deficits based on the difference between production and consumption of crop calories. The crop calorie consumption consisted of human vegetal product intake and crop-based feed provided to livestock. We estimated vegetal product consumption (V_x) in a $5'$ raster grid by multiplying countrywide per capita vegetal product intake [3] with the gridded population data [4] for the year 2000. Before that, we aggregated the gridded population data in $2.5'$ resolution to $5'$ by summing up population counts. Moreover, for those countries with the total food consumption of less than 2,200 kcal/cap/day we adjusted the vegetal products consumption to meet the average moderate calorie diet of 2,200 kcal/cap/day [5]. For crop-based feed consumption, we used the gridded feed data (F_x) [6]. On CMR level, we estimated crop calorie deficits (Da_z and Dh_z) based on the current and the potential crop calorie production (Ca_z and Ch_z) respectively, as represented by equations 5 and 6.

$$Da_z = \left(\sum_{x \in z} (V_x + F_x)\right) / Ca_z \tag{5}$$

$$Dh_z = \left(\sum_{x \in z} (V_x + F_x)\right) / Ch_z \tag{6}$$

S1.3 Text Clustering Regions

In the next step, we classify regions into the six groups based on prevalence and depth of crop production gaps and crop calorie deficits. The following procedures are used:

1. If $Da_z \leq 1$, the regions achieved FSS. We considered these regions as capable of producing enough crop calories to meet their consumption demand.

2. Afterwards, we investigated the regions that are capable of producing enough crop calories by achieving the high-input potential yields. For this, we distinguished arid and humid regions as agricultural management varies across agro-climatic zones. Hence, for the remaining regions, if $Dh_z \leq 1$ and falls in one of the hyper-arid, arid, dry semi-arid and moist semi-arid zones, we marked it $FSS_{hi,ar}$. However, if it falls in one of the sub-humid, humid and per-humid zones, we marked it $FSS_{hi,hm}$.

3. Then, if the remaining regions with crop calorie production gap (i.e., $Gh_z < 1$) fall in one of the hyper-arid, arid, dry semi-arid and moist semi-arid zones, we marked them $ICP_{hi,ar}$. However, if they fall in one of the sub-humid, humid and per-humid zones, marked them $ICP_{hi,hm}$. These are regions capable of producing more crop calories by achieving high-input potential yields but aren't food self-sufficient.

4. The final step consists of marking the entire remaining regions that are crop deficits as crop insufficient (CIS). These are the regions which almost achieved their potential crop calorie production and are dependent on trade to meet feed and food demand.

S1.4 Text Scenarios

To analyze scenarios for 2050, we considered changes in population [7] and dietary patterns [5] that drives the future food and feed demand [6], and progress on closing crop yield gaps that influences the future food and feed supply.

The first one (scenario A) is a baseline scenario where the dietary pattern of a country stays the same as in the year 2000 but population changes for all countries. This scenario provides results for a low bound. Moreover, for those countries with the total food consumption of less than 2,200 kcal/cap/day, we maintained a minimum calorie intake of 2,200 kcal/cap/day as described above. We used gridded population for 2050 [8] that was downscaled based on the mid-range population scenario from the United Nations [7] and gridded feed calorie demand for 2050 [6]. The gridded feed calorie demand is based on similar scenario accounting for population growth with constant dietary pattern.

The second scenario (scenario B) takes into account country-specific changes in dietary patterns in addition to the population growth, however, maintaining a minimum calorie intake of 2,535 kcal/cap/day, representing an average of high calorie diets [5]. The future per capita food demand by country is estimated up to 2050 based on observed exponential relationships between per capita food intake and the Human Development Index (HDI) [5], using the HDI extrapolation based on logistic regression [9]. This scenario provides results for an upper bound based on changes in dietary patterns and population. Additionally, we used data on gridded feed calorie demand for 2050 considering similar changes in population and dietary patterns [6].

We carried out scenario analyses to identify regions where closing the production gaps matters to ensure the future food self-sufficiency using the methods described in S1 Text. The estimation of future crop calorie production needs to account for complex influences of population change, climate change and technological progress since the year 2000. However, we kept our analysis simple by considering the crop calorie production in the year 2000 and the high input potential crop calorie production with a constant climate. But we took into account loss in crop production due to an increase in population leading to an expansion of build-up land encroaching on cultivated land based on Pradhan et al. [8]. Crop production changes due to an increase in build-up land and population was kept the same for both scenarios.

References

1. IIASA/FAO. Global Agro-ecological Zones (GAEZ v3.0). IIASA, Laxenburg and FAO, Rome: IIASA/FAO; 2012. Available from: `http://webarchive.iiasa.ac.at/Research/LUC/GAEZv3.0/`.

2. FAO. Food balance sheets: A handbook. Rome: FAO; 2001.

3. FAO. FAOSTAT- FAO Statistical Databases: Agriculture, Fisheries, Forestry, Nutrition. Rome: Food and Agriculture Organization of the United Nations; 2012. Available from: `http://faostat.fao.org/`.

4. CIESIN/IFPRI/CIAT. Global Rural-Urban Mapping Project, Version 1 (GRUMPv1): Population Count Grid. Palisades, NY: NASA Socioeconomic Data and Applications Center (SEDAC); 2011. Available from: `http://dx.doi.org/10.7927/H4VT1Q1H`.

5. Pradhan P, Reusser DE, Kropp JP. Embodied Greenhouse Gas Emissions in Diets. PLOS ONE. 2013;8(5):e62228.

6. Pradhan P, Lüdeke MK, Reusser DE, Kropp JP. Embodied crop calories in animal products. Environ Res Lett. 2013;8(4):044044.

7. United Nations. World Population Prospect: The 2010 Revision - Highlights and Advance Tables. New York: UN; 2011.

8. Pradhan P, Lüdeke MKB, Reusser DE, Kropp JP. Food self-sufficiency across scales: How local can we go? Environ Sci Technol. 2014;48(16):9463–9470.

9. Costa L, Rybski D, Kropp JP. A Human Development Framework for CO_2 Reductions. PLOS ONE. 2011;6(12):e29262.
