# Supplementary material for: Closing Yield Gaps: How Sustainable Can We Be?
Source: PLoS One. 2015 Jun 17;10(6):e0129487. doi: 10.1371/journal.pone.0129487 (PMC4470636; doi:10.1371/journal.pone.0129487)
Supplement: S2 Text — (PDF) [file pone.0129487.s002.pdf]

S2 Text Identification of Management Strategies

A number of biophysical and socioeconomic factors have an effect on the yield gaps [1, 2]. In general, such biophysical factors are: nutrient imbalances, water scarcity, climate variability, suboptimal planting, weed pressure, insect and disease damage, inferior seed quality, and climatic and edaphic workability constraints. Socioeconomic factors are: profit maximization, credit availability, limited labor supply, knowledge on best practices, accessibility to market, farm size, land tenure and extension services. Data on spatial distribution of such factors are limited. Hence, we used the GAEZv3.0 model [3] to identify spatial distribution of constraints that the above factors may exhibit. With this, we covered most biophysical factors, however, socioeconomic factors are limitedly represented and based on few indicators.

We started the analysis looking at agro-climatic constraints that represent climate related yield losses due to pests, diseases, weeds, and workability. GAEZv3.0's Module III provides spatial distribution of agro-climatic constraints factor (cf) in percentage in a $5'$ raster grid for low (l) and high (h) input farming by crops, which were derived based on climatic conditions. The values of cf represent attainable percentage of the constraint free crop yields considering yield losses due to agro-climatic constraints for a specific level of agricultural inputs and management conditions. A low-input farming system is largely subsistence labor intensive agriculture, lacking application of nutrients and agro-chemicals, traditional management with minimum conservation measures and a high-input farming system is mainly commercial mechanized agriculture with optimum application of nutrients, and agro-chemicals [3]. This factor is related to yield reduction due to pests, diseases, weeds, and workability, of which the first three could be overcome by improved pest management, including application of agro-chemicals. However, climate related soil workability does not improve with high-input farming. We calculated the difference (df) between the agro-climatic constraints factor for low and high input farming for a crop to identify regions where the constraints could be overcome by shifting from low to high input farming (equation 7). We used crops (j) in two crop groups (cereals and roots-tubers) to estimate weighted difference (dF) based on their respective harvested area (ha) (equation 8).

$$df = cf_h - cf_l \tag{7}$$

$$dF = \left(\sum_{j=1}^{n} \left(df^j \times ha^j \right) \right) / \left(\sum_{j=1}^{n} ha^j \right) \tag{8}$$

GAEZv3.0's Module IV carries out edaphic assessment and simulated yield reduction due to soil and terrain limitations [3]. We used data on soil limitations from GAEZv3.0 to identify regions where there is a need for soil quality management to bridge the yield gap. GAEZv3.0 differentiates soil qualities into seven types: nutrient availability, nutrient retention capacity, rooting conditions, soil drainage associated with oxygen availability to roots, excess salts, toxicity and workability. Constraints related to these soil qualities are classified mainly into four categories: no or slight constraints, moderate constraints, severe constraints, and very severe constraints. Among the seven soil qualities, constraints related to three of them (rooting conditions, excess salts, and toxicity) are difficult to overcome by high-input farming. Additionally, nutrient supply is essential for achieving high yield, which we accounted separately and elaborated in the next section. Therefore, we considered the remaining three soil quality factors (nutrient retention capacity, soil drainage, and soil workability) for further analysis. Subsequently, we identified regions where constraints related to one or multiple of these qualities are moderate to very severe. These are regions where improved management of one or multiple of these soil qualities would enhance crop yields.

Secondly, we attempted to capture the socioeconomic factors that play important roles in closing yield gaps based on two indicators: yield variability and travel time to the nearest market. The yield variability due to weather conditions might make farmers reluctant to take risks in terms of input applications.

GAEZv3.0 also provides data on coefficient of variation of agro-climatically attainable yields for the baseline period of 1961–1990 [3]. We used this data for crops (j) in two crop groups (cereals and roots-tubers) to estimate weighted yield variations (CV) based on irrigated (i) and rain-fed (r) harvested area (ha), and identified regions with high overall year-to-year yield variations (equation 9). Travel time to the nearest market is also an important factor to support agricultural productivity in two ways. Firstly, it determines farmers' accessibility to agricultural inputs. Secondly, it influences market accessibility for vending agricultural products. Consequently, we used spatially explicit accessibility data presenting travel time to the nearest market with a population of around 50,000 [3] to identify regions with different connectivity to markets.

$$CV = \left(\sum_{j=1}^{n} \left(cv^j \times ha_r^j \right) \right) / \left(\sum_{j=1}^{n} \left(ha_r^j + ha_i^j \right) \right) \tag{9}$$

Lastly, we determined regions with similar constraint compositions from the prevalence of the four constraints mentioned above (soil related constraints, weather induced yield variability, agro-climate related pest, disease, and weed constraints, as well as market accessibility). The composite constraints factors used are as follows:

- weighted difference between agro-climatic constraints factor (dF) for low-input and high-input farming larger than 5%,
- any of the above mentioned three soil quality constraints,
- weighted yield variation (CV) larger than 20%, and
- travel time to nearest market greater than 6 hours

Beyond the application of nutrients and the use of improved cultivars, there is a need to include other specific agricultural inputs, management, and socioeconomic infrastructure that tackles the constraints described above to close yield gaps. Details on such specific input and management interventions are provided in the main text. Location specific input and agricultural management strategies depend on prevalence of one or more of the above constraints. For simplicity, we designed agriculture management strategies such that they aim to overcome and reduce constraints causing yield gaps.

References

1. Lobell DB, Cassman KG, Field CB. Crop yield gaps: their importance, magnitudes, and causes. Annu Rev Environ Resour. 2009;34(1):179.

2. Fischer TR, Byerlee D, Edmeades GO. Can technology deliver on the yield challenge to 2050? In: Conforti P, editor. Looking ahead in world food and agriculture: perspectives to 2050. Rome: FAO; 2011. p. 389–462.

3. IIASA/FAO. Global Agro-ecological Zones (GAEZ v3.0). IIASA, Laxenburg and FAO, Rome: IIASA/FAO; 2012. Available from: `http://webarchive.iiasa.ac.at/Research/LUC/GAEZv3.0/`.
