# Supplementary material for: Closing Yield Gaps: How Sustainable Can We Be?
Source: PLoS One. 2015 Jun 17;10(6):e0129487. doi: 10.1371/journal.pone.0129487 (PMC4470636; doi:10.1371/journal.pone.0129487)
Supplement: S3 Text — (PDF) [file pone.0129487.s003.pdf]

# S3 Text Nutrients Required

We further estimated the amount of three macro-nutrients required (N, $P_2O_5$, and $K_2O$) to achieve the potential yields. To obtain a sustainable crop yield superior to the low-input yields, nutrient application is needed in addition to natural nutrient regeneration. For quantifying the required nutrients, we initially calculated crop nutrient uptake in yield and in residue. We considered differences between yields and residues of the 16 crop types from GAEZv3.0 for high and low inputs, taking into account their fallow period requirements. In low-input farming, the fallow period requirement is longer than that for high-input due to need for natural nutrient regeneration that is substituted by fertilizer application in high-input agriculture. GAEZv3.0 provides crop-specific fallow period requirements ($f_{i,j}$: for crop j in location i) for high ($h$) and low ($l$) inputs by soil type and by climatic condition. We obtained fallow factor ($\kappa_{i,j}$) for high and low inputs using the respective fallow period requirements as presented by equation 10. Residue for a crop type ($R_{i,j}$) was estimated based on its yield ($Y_{i,j}$) and harvest index ($hi_j$) [1] using equation 11. Harvest index is represented by the weight of harvested crop as a percentage of the total plant weight.

$$\kappa_{i,j} = \frac{(100 - f_{i,j})}{100} \tag{10}$$

$$R_{i,j} = (1 - hi_j) \times Y_{i,j} \tag{11}$$

Afterwards, multiplying the differences in crop yields and residues by the crop specific nutrient uptake per tonne of yield ($Fc_j^k$: for nutrient type $k$) and per tonne of residue ($Fr_j^k$) respectively (Table S1), we estimated the amount of nutrient uptake in the crop yield and the crop residue as shown by equation 12 and 13. GAEZv3.0 provides information on the current and the potential crop yields for two types of water supply (irrigated and rain-fed) and the potential yields for three input levels (low, intermediate, and high). Since data on irrigated crop yields is provided only for intermediate and high input levels, we considered the differences between crop yield and crop residue based on high and intermediate inputs for irrigated cultivated land, instead of the low input ones in equations 12 and 13.

$$Fc_{i,j}^k = \left(\kappa_{i,j}^h \times Y_{i,j}^h - \kappa_{ij}^l \times Y_{i,j}^l\right) \tag{12}$$

$$Fr_{i,j}^k = \left(\kappa_{i,j}^h \times R_{i,j}^h - \kappa_{ij}^l \times R_{i,j}^l\right) \tag{13}$$

In the next step, we obtained total nutrient uptake in crop yields and residues summing up the individual nutrient uptake for the 16 crop types in GAEZv3.0 excluding olive, oil palm, cotton, cash crop 1, cash crop 2, fodder, and residual as presented by equations 14. For this, we considered crop yields in both irrigated ($w$) and rain-fed ($r$) cultivated land and respective harvest area ($ha$). Sum up of nutrient uptake in yields and residues provides total additional nutrient uptake ($NF_i^k$) by the high input crop production compared to the low input ones.

$$NF_i^k = \sum_{j=1}^{16} \left(Fc_{i,j}^{k,r} \times ha_{i,j}^r + Fc_{ij}^{k,w} \times ha_{i,j}^w\right) + \sum_{j=1}^{16} \left(Fr_{i,j}^{k,r} \times ha_{i,j}^r + Fr_{ij}^{k,w} \times ha_{i,j}^w\right) \tag{14}$$

Finally, the total nutrients required were estimated considering nutrient removal and nutrient losses due to leaching and volatilization. Here we assumed that both crop products (e.g., grain) and residues (e.g., straw) are removed from the field. Hence, nutrient removal that has to be replenished by nutrients (organic or chemical fertilizer), is equal to nutrient uptake in crop yields and residues. Additionally, nutrient losses depend on nutrient type and its application efficiencies. Therefore, we considered a parameter inverse of the fertilizer application efficiency $\tau_i^k$ that may vary spatially, to estimate the total nutrient required (equation 15).

$$GF_i^k = NF_i^k \times \tau_i^k \tag{15}$$

# References

1. IIASA/FAO. Global Agro-ecological Zones (GAEZ v3.0). IIASA, Laxenburg and FAO, Rome: IIASA/FAO; 2012. Available from: http://webarchive.iiasa.ac.at/Research/LUC/GAEZv3.0/.
