# Supplementary figures and images for: Closing Yield Gaps: How Sustainable Can We Be?
Source: PLoS One. 2015 Jun 17;10(6):e0129487. doi: 10.1371/journal.pone.0129487 (PMC4470636; doi:10.1371/journal.pone.0129487)

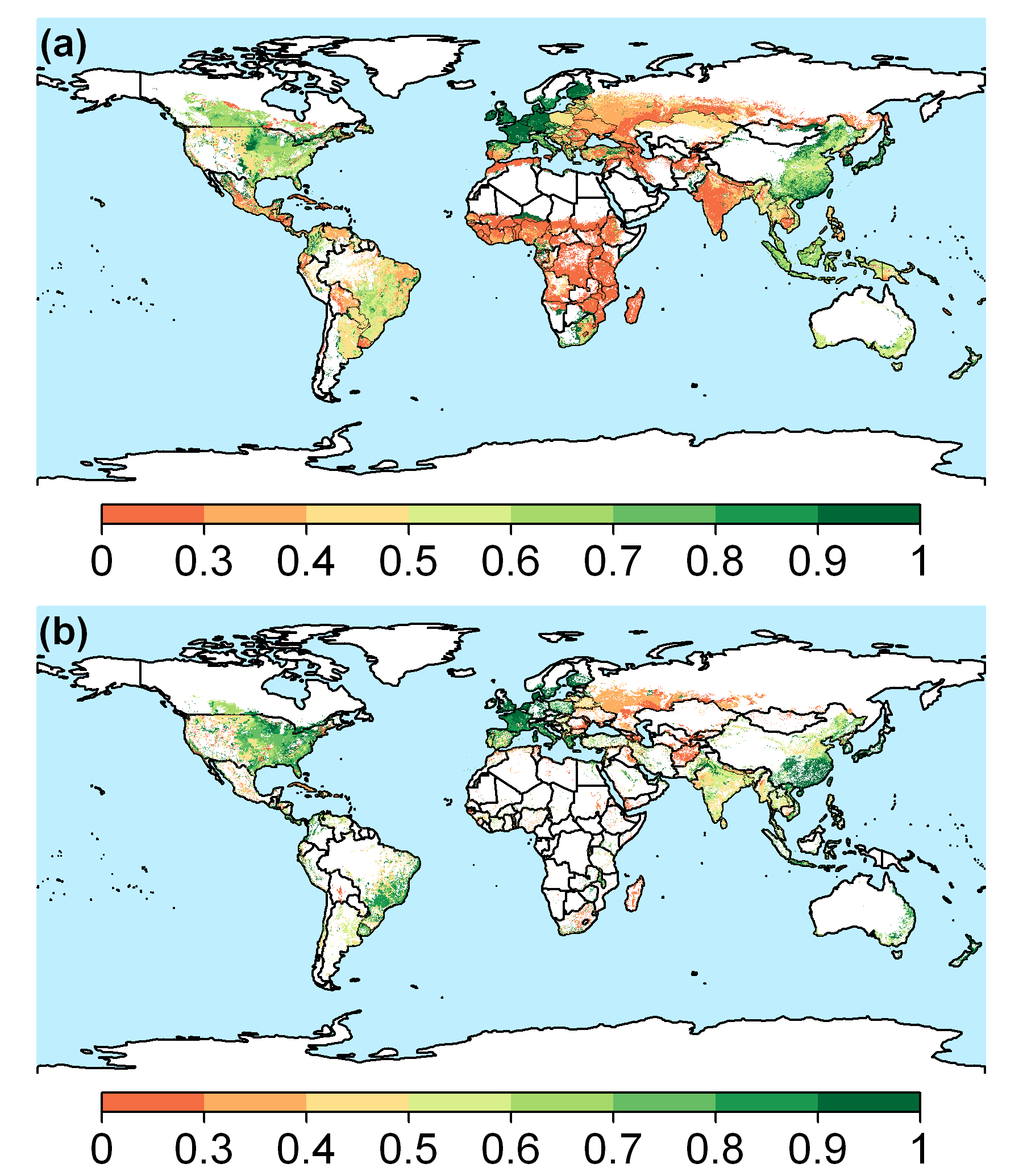

Supplement: S1 Fig — (a) rain-fed cultivated land, and (b) irrigated cultivated land. A ratio of 1 represents regions that have achieved their high-input crop calorie production. (TIF) [file pone.0129487.s004.tif]

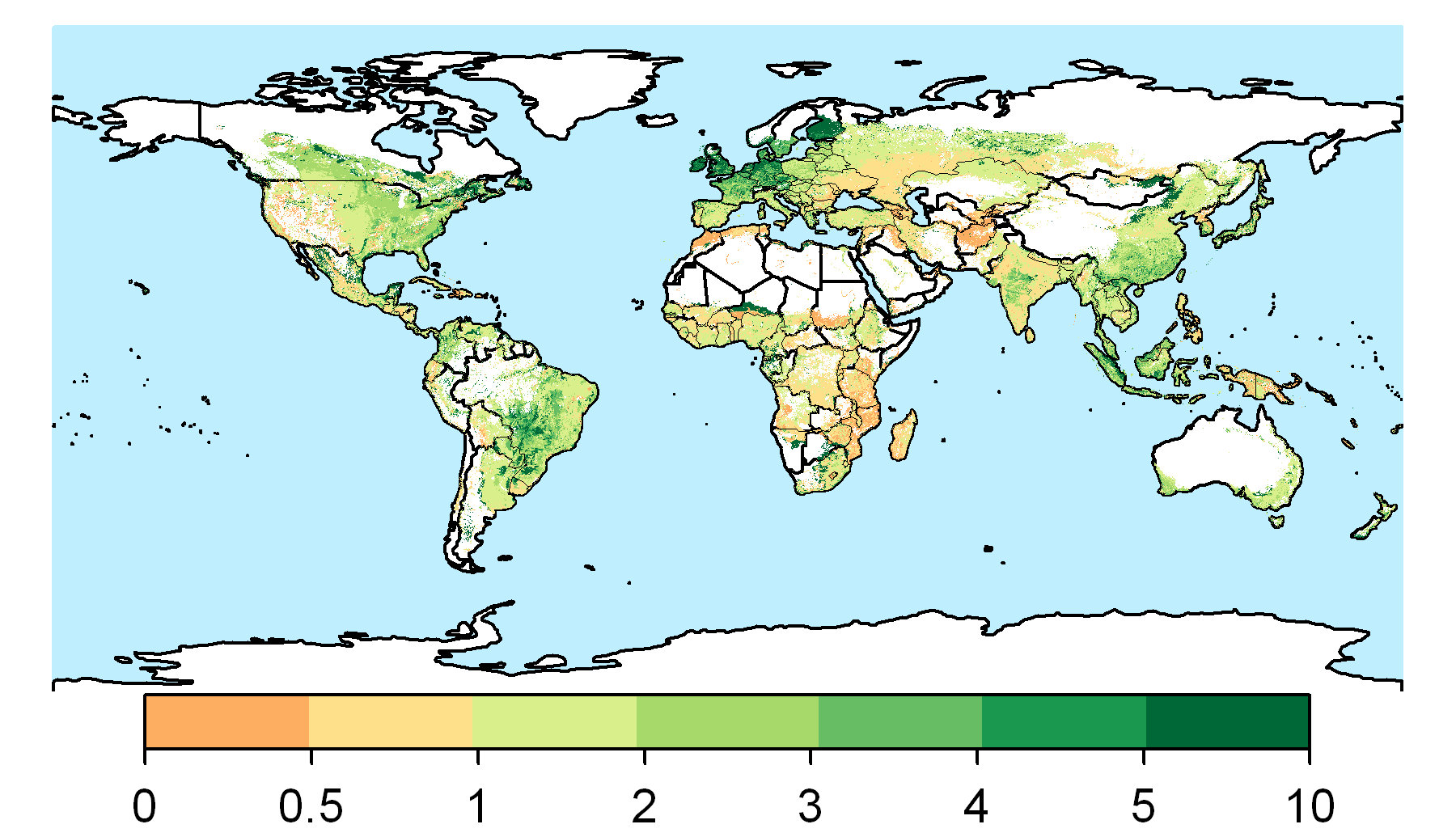

Supplement: S2 Fig — The values greater than 1 represent regions with the current crop calorie production larger than the low-input calorie production. (TIF) [file pone.0129487.s005.tif]

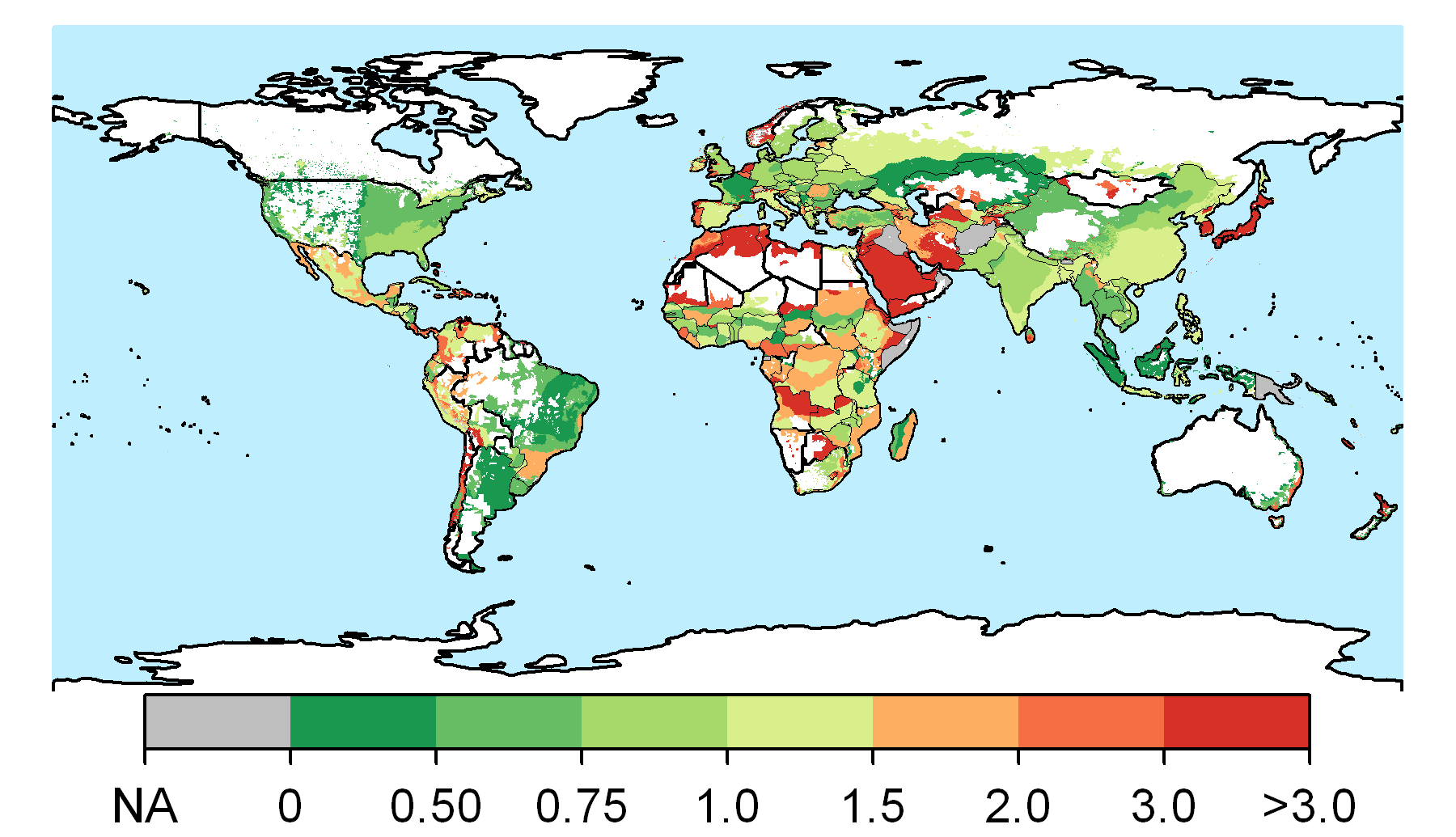

Supplement: S3 Fig — The values greater than 1 represent regions with crop calorie consumption greater than crop calorie production. Since agricultural production constraints and agricultural management vary with agro-climatic conditions, the results are presented by country moisture regime going beyond national scales. NA represents regions with missing data. (TIF) [file pone.0129487.s006.tif]

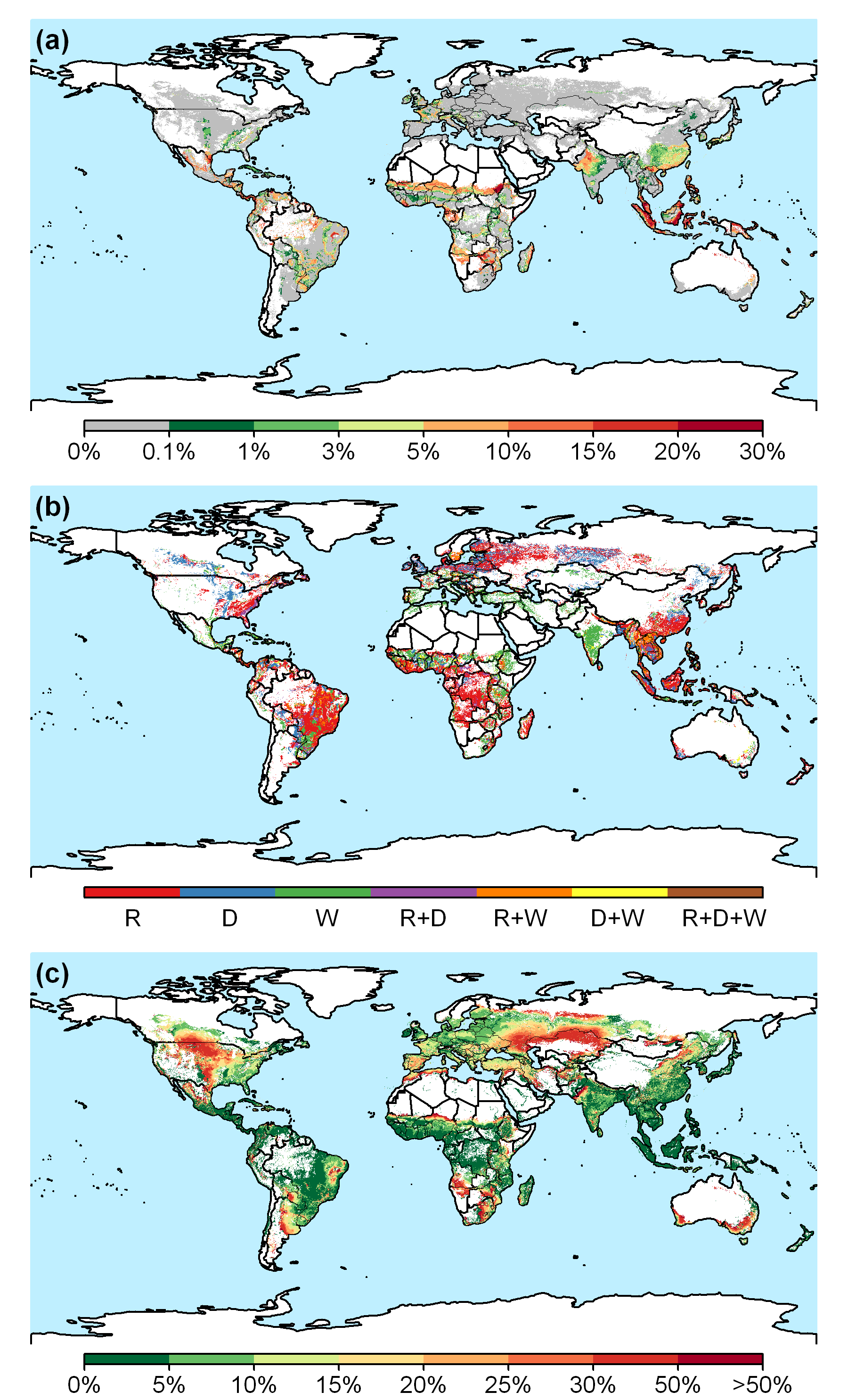

Supplement: S4 Fig — (a) weighted difference between agro-climatic constraints factor in percentage for low-input and high-input farming, (b) location specific most severe soil quality constraints based on three soil qualities (nutrient retention capacity (R), soil drainage (D), and soil workability (W)), and (c) weighted coefficient of variation of agro-climatic yields in percentage. (TIF) [file pone.0129487.s007.tif]

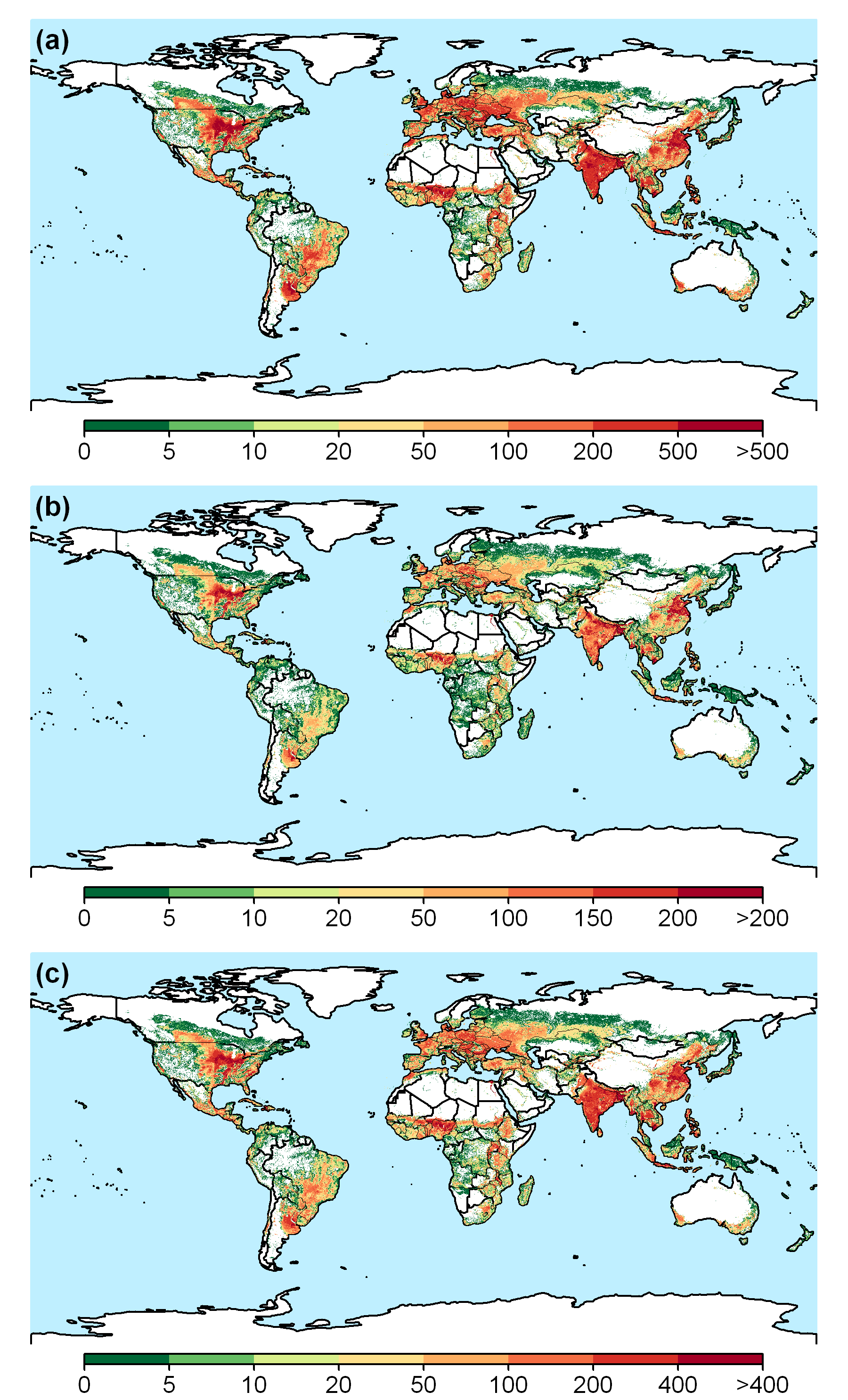

Supplement: S5 Fig — (a) nitrogen—N total nutrients, (b) phosphate—P2O5 total nutrients, and (c) potash—K2O total nutrients. (TIF) [file pone.0129487.s008.tif]
