# Supplementary material for: Closing Yield Gaps: How Sustainable Can We Be?
Source: PLoS One. 2015 Jun 17;10(6):e0129487. doi: 10.1371/journal.pone.0129487 (PMC4470636; doi:10.1371/journal.pone.0129487)
Supplement: S1 Table — (PDF) [file pone.0129487.s009.pdf]

S1 Table. List of crop types for which data on harvest area (H^j) (second column) and crops for which data on potential yield (Y^k) (thrid column) is provided by GAEZv3.0 [1] along with their nutritive factors (f^j) [2], conversion factor (c^k) for harvested weight to dry weight [1] and nutrient uptakes from various sources compiled by authors [3–5].

No.	Crop Type	Crop	Nutritive factors (kcal/100gm)	Conversion factor	N (kg/t)		P_2O_5 (kg/t)		K_2O (kg/t)	
					yield	residue	yield	residue	yield	residue
1	Maize	Maize	356	0.87	12.0	8.0	6.3	2.9	4.5	20.0
2	Millet	Foxtail millet	340	0.9	28.0	7.7	8.0	2.2	8.0	20.0
		Pearl millet								
3	Other cereals	Barley	340	0.888	21.0	6.5	8.3	2.6	6.7	20.0
		Buck wheat			14.0	12.6	5.0	1.6	4.4	13.9
		Oat			24.0	6.6	8.8	3.2	5.9	19.0
		Rye			25.0	6.0	8.2	1.5	5.5	11.0
4	Rice	Dryland rice	280	0.9	13.0	8.3	6.7	2.7	3.6	21.0
		Wetland rice		0.875						
5	Sorghum	Sorghum	332	0.88	13.0	14.0	7.8	4.2	5.4	21.0
6	Wheat	Wheat	334	0.875	22.0	7.6	8.8	1.9	5.2	15.0
7	Banana & coconut	Banana	122	0.35						
		Coconut		0.175						
8	Groundnut	Groundnut	414	0.67	6.4	3.6	6.9	3.2	12.0	12.0
9	Oil palm	Oil palm	158	0.225						
10	Olive	Olive	175	0.22						
11	Rapeseed	Rapeseed	494	0.9	38.0	6.8	24.0	2.4	40.0	11.4
12	Soybean	Soybean	335	0.9	55.0	20.0	12.0	4.4	20.0	19.0
13	Sunflower	Sunflower	308	0.9	27.0	12.0	9.7	1.0	9.0	17.0
14	Pulses	Chickpea	340	1	38.0	22.3	4.1	4.6	11.2	10.9
		Cowpea			27.2	27.2	2.7	2.7	18.2	18.2
		Drypea			38.0	22.3	4.1	4.6	11.2	10.9
		Gram			50.0	27.2	13.0	6.0	15.0	18.1
		Phaseolus bean			50.0	27.2	13.0	6.0	15.0	18.1
		Pigeonpea			38.0	22.3	4.1	4.6	11.2	10.9
15	Cassava & yam	Cassava	98.7	0.35	3.2	2.0	0.9	1.1	4.8	3.0
		Yam & cocoyam								
16	Potato	Sweet potato	79.5	0.275	3.2	2.0	1.2	0.5	5.5	3.0
		White potato								
17	Sugar beet	Sugar beet	70	0.14	1.9	3.7	1.1	2.0	3.7	10.0
18	Sugarcane	Sugarcane	30	0.1	1.0	1.5	0.7	0.3	1.8	1.2
19	Vegetables	Cabbage	22	0.15	3.3	3.3	0.9	0.9	3.2	3.2
		Carrot			1.9	1.2	0.9	0.2	3.0	3.1
		Onion			2.7	3.0	1.4	7.5	2.3	4.4
		Tomato			1.3	18.2	0.5	4.2	2.9	36.0

References

1. IIASA/FAO. Global Agro-ecological Zones (GAEZ v3.0). IIASA, Laxenburg and FAO, Rome: IIASA/FAO; 2012. Available from: http://webarchive.iiasa.ac.at/Research/LUC/GAEZv3.0/.

2. FAO. Food balance sheets: A handbook. Rome: FAO; 2001.

3. IPNI. IPNI Estimates of Nutrient Uptake and Removal; 2012. Available from: http://www.ipni.net/article/IPNI-3296.

4. NRCS. Amount of plant nutrients contained in crops. New Mexico: US Department of Agriculture; 2000.

5. MSU. Crop removal charts - Field Crops Team; 1992. Available from: http://fieldcrop.msu.edu/uploads/documents/crop_remova_charts.pdf.
