# Supplementary material for: Closing Yield Gaps: How Sustainable Can We Be?
Source: PLoS One. 2015 Jun 17;10(6):e0129487. doi: 10.1371/journal.pone.0129487 (PMC4470636; doi:10.1371/journal.pone.0129487)
Supplement: S2 Table — The strategies consist of soil quality management (S), managing accessibility to markets (A), weather induced yield variability management (V), and management of pests, diseases, and weeds (P). The different management strategies can have combinations of the individual elements (F, S, A, V, and P). (PDF) [file pone.0129487.s010.pdf]

S2 Table. Regional overview on area of rain-fed cultivated land where different inputs and management strategies in addition to adequate fertilizer application (F) are required to close the yield gaps. The strategies consist of soil quality management (S), managing accessibility to markets (A), weather induced yield variability management (V), and management of pests, diseases, and weeds (P). The different management strategies can have combinations of the individual elements (F, S, A, V, and P).

**Regions**	**F**	**S**	**A**	**AS**	**V**	**VS**	**VA**	**VAS**	**P**	**PS**	**PA**	**PAS**	**PV**	**PVS**	**PVA**	**PVAS**	**Total**
	in million hectares																
**Africa**																	
East Africa	6.35	25.34	4.23	12.09	1.16	5.53	0.83	2.36	0.48	2.05	0.13	0.5	0.07	0.11	0.01	0.03	61.27
Middle Africa	0.98	15.84	0.67	13.21	0.07	1.72	0.05	2.15	0.07	1.34	0.03	1.37	0	0.03	0	0	37.53
North Africa	4.17	7.68	1.45	4.02	3.52	5.06	0.08	0.56	0.03	0.05	0.14	0.19	0	0	0	0	26.95
South Africa	1.4	3.49	0.4	0.98	2.31	5.55	0.32	1.31	0	0	0	0	0	0	0	0	15.76
West Africa	8.81	56.91	1.7	10.29	0.04	1.83	0.05	1.47	0.12	2.13	0.01	0.49	0	0	0	0	83.85
**America**																	
Caribbean	1.99	3.11	0.06	0.05	0.08	0.17	0.01	0.02	0.04	0.08	0	0	0	0	0	0	5.61
Central America	7.1	9.08	1.12	1.58	0.73	1.01	0.08	0.19	2.8	3.71	0.48	0.69	0.06	0.04	0	0.02	28.69
North America	42.75	40.58	4.42	2.79	77.04	21.28	3.28	0.55	0.49	1.42	0.01	0.05	0.01	0.02	0	0	194.69
South America	17.56	53.17	2.31	10.35	7.61	5.99	1.17	1.36	1.4	11.47	0.47	2.13	0.05	0.48	0.02	0.08	115.62
**Asia**																	
Central Asia	0.84	0.47	0.14	0.06	11.62	2.62	7.9	2.65	0	0	0	0	0	0	0	0	26.30
East Asia	32.98	26.73	6.28	4.51	6.09	2.29	1.63	0.32	1.37	7.03	0.17	0.37	0.02	0.02	0.01	0.01	89.83
South Asia	39.7	70.09	2.26	4.38	7.11	9.67	2.01	1.68	0.34	0.95	0.02	0.09	0	0.02	0.01	0.03	138.36
S.-East Asia	5.2	28.61	3.25	8.83	0.08	0.14	0.03	0.17	2.88	19.87	1.01	10.56	0.02	0.03	0.02	0.12	80.82
West Asia	13.38	8.42	0.49	0.38	3.79	2.38	0.13	0.1	0.03	0.06	0	0	0.01	0	0	0	29.17
**Europe**																	
East Europe	54.09	41.67	1.42	1.3	77.19	12.12	2.84	1.18	0.04	0.04	0	0	0.01	0	0	0	191.90
North Europe	3.87	12.1	0.03	0.09	0.03	0.13	0.01	0.03	0.33	0.68	0	0	0	0	0	0	17.30
South Europe	10.87	8.8	0.24	0.1	6.05	4.73	0.17	0.05	0.97	0.77	0.01	0.01	0.02	0.01	0	0	32.80
West Europe	12.63	12.35	0.02	0.02	0.56	0.47	0	0	2.6	2.32	0	0	0.23	0.08	0	0	31.28
**Oceania**																	
Australia & New Zealand	5.16	5.17	0.54	0.96	15.14	12.28	1.69	2.42	0.18	0.26	0	0.01	0	0	0	0	43.81
**World**	**269.82**	**429.6**	**31.01**	**75.98**	**220.23**	**94.95**	**22.29**	**18.56**	**14.18**	**54.23**	**2.49**	**16.47**	**0.51**	**0.86**	**0.06**	**0.3**	**1,251.54**
