# Supplementary material for: Closing Yield Gaps: How Sustainable Can We Be?
Source: PLoS One. 2015 Jun 17;10(6):e0129487. doi: 10.1371/journal.pone.0129487 (PMC4470636; doi:10.1371/journal.pone.0129487)
Supplement: S3 Table — (PDF) [file pone.0129487.s011.pdf]

S3 Table. Regional overview on additional amount of macro-nutrients (N, P_2O_5, and K_2O) uptake by crop yields (Y) and crop residues (R) by attaining high input potential yields compared to that with low input yields, and regional overview on the amount of fertilizers applied in the year 2010.

Regions	**N** uptake & applied (1000 tonnes/year)			**P_20_5** uptake & applied (1000 tonnes/year)			**K_20** uptake & applied (1000 tonnes/year)		
	yield (Y)	residue (R)	applied (A)	yield (Y)	residue (R)	applied (A)	yield (Y)	residue (R)	applied (A)
**Africa**									
East Africa	2,455	970	581	294	967	330	1,077	1,645	90
Middle Africa	557	232	21	80	233	9	321	418	15
North Africa	1,494	533	1,723	160	648	523	507	945	76
South Africa	357	124	398	40	167	162	131	270	106
West Africa	3,450	1,484	287	450	1,360	130	1,479	2,441	97
**America**									
Caribbean	134	65	179	20	59	51	77	105	43
Central America	1,022	445	1,563	137	458	290	390	805	323
North America	11,976	3,216	13,472	896	4,195	4,503	4,221	5,387	4,585
South America	7,273	2,131	5,651	555	2,270	4,,721	2,693	3,102	4,676
**Asia**									
Central Asia	962	195	833	52	381	127	250	387	37
East Asia	8,574	2,508	35,752	803	3,688	17,504	3,367	5,110	5,766
South Asia	11,377	3,888	21,343	1,096	4,505	9,616	3,865	6,819	3,936
S.-East Asia	3,469	1,381	7,117	441	1,629	1,814	1,179	3,088	2,746
West Asia	1,895	464	1,987	127	724	737	527	852	149
**Europe**									
East Europe	9,697	2,261	5,002	600	3,739	1,580	2,883	4,417	1,819
North Europe	1,083	246	2,224	67	419	498	344	469	644
South Europe	1,479	401	2,033	117	594	864	459	804	789
West Europe	2,234	510	4,213	143	909	653	788	981	978
**Oceania**									
Australia & New Zealand	986	228	1,487	60	397	1,325	294	418	221
**World**	**70,513**	**21,297**	**105,890**	**6,141**	**27,356**	**45,442**	**24,874**	**38,492**	**27,112**
